# Limited Detectability of Network Functional Alterations in a Tauopathy Model Using Mouse Primary Cortical Cultures

**DOI:** 10.3390/biomedicines14081670

**Published:** 2026-07-24

**Authors:** Clara F. López-León, Julia Sala-Jarque, José Antonio del Río, Jordi Soriano

**Affiliations:** 1 Departament de Física de la Matèria Condensada, Universitat de Barcelona, 08028 Barcelona, Spain; 2 Universitat de Barcelona Institute of Complex Systems (UBICS), Universitat de Barcelona, 08028 Barcelona, Spain; 3 Department of Molecular, Cellular, Developmental Biology, Neuroscience Research Institute, University of California, Santa Barbara, CA 93106, USA; jsalajarque@ucsb.edu; 4 Institute for Bioengineering of Catalonia (IBEC), The Barcelona Institute of Science and Technology, 08028 Barcelona, Spain; jadelrio@ub.edu; 5 Centro de Investigación Biomédica en Red sobre Enfermedades Neurodegenerativas (CIBERNED), 28031 Madrid, Spain; 6 Department of Cell Biology, Physiology and Immunology, Faculty of Biology, Universitat de Barcelona, 08028 Barcelona, Spain; 7 Institute of Neuroscience, University of Barcelona, 08028 Barcelona, Spain

**Keywords:** neuronal cultures, fluorescence calcium imaging, tauopathies, functional connectivity

## Abstract

**Background:** Tauopathies are neurodegenerative disorders characterized by the abnormal hyperphosphorylation and aggregation of the microtubule-associated protein tau, leading to disrupted neuronal connectivity and progressive brain dysfunction. Despite their clinical relevance, most in vitro models have focused primarily on molecular and cellular aspects, with limited emphasis on alterations in network dynamics and functional connectivity. **Methods:** We developed an in vitro tauopathy model based on mouse primary neuronal cultures, enabling the investigation of network-level alterations under controlled conditions. We compared three experimental groups: untreated control cultures, cultures exposed to extracellular wild-type tau, and cultures treated with pathological tau (pTau) isolated from the sarkosyl-insoluble fraction of P301S (+/−) transgenic mice. To increase susceptibility to tau-induced pathology, all conditions were additionally transduced with adeno-associated viral vectors encoding human P301L tau. To quantify for damage, spontaneous neuronal activity was monitored throughout network maturation—from day in vitro (DIV) 7 to 16—using fluorescence calcium imaging, and multiple metrics describing network dynamics and functional organization were compared at DIV 12. **Results:** We observed that exposure to pTau did not induce overt cytotoxicity or major disruptions in global network dynamics, although a mild increase in network bursting activity was observed. Longitudinal analysis of network maturation further revealed largely similar developmental trajectories across experimental groups, with only subtle and persistent differences in bursting-related activity in pTau-treated cultures. **Conclusions:** We propose that the early developmental stage of the cultures, together with the intrinsic bursting and ongoing synaptic plasticity of primary neuronal networks, masks subtle pathological effects. This may limit the sensitivity of two-dimensional in vitro systems to detect network-level dysfunction, suggesting that more mature or structurally complex models, such as brain organoids, may be required to reveal robust functional deficits.

## 1. Introduction

Tau is a microtubule-associated protein essential for stabilizing neuronal structure and supporting axonal transport [1]. Under disease conditions, tau becomes hyperphosphorylated, detaches from microtubules, and aggregates into insoluble filaments and neurofibrillary tangles known as *tau aggregates*. These pathological forms of tau (pTau) disrupt neuronal communication and impair synaptic plasticity [1], overall contributing to cognitive decline across a range of tauopathies [2,3], including Alzheimer’s disease (AD) [4,5] and frontotemporal lobar degeneration [6]. Although tau aggregation is a shared hallmark, the affected cell types, regional distribution, and aggregate characteristics vary substantially across diseases [7,8].

A shared characteristic of tauopathies is the affectation of molecular mechanisms that scale up to altered neuronal excitability and network organization. Insoluble tau filaments can disturb calcium homeostasis [9,10], compromise mitochondrial function [11,12], and promote hyperactivity in vulnerable neuronal populations [13]. In this context, it was proposed that aberrant network hyperactivity could arise from compensatory responses to degeneration [14,15], but these same mechanisms may accelerate tau pathology, creating a harmful feedback loop. Despite extensive research, the precise mechanisms driving pTau progression remain unresolved, though a prominent hypothesis proposes that tau spreads in a prion-like manner [16,17] through templated misfolding and trans-synaptic transfer, a process known as *seeding* [18].

To investigate these processes under controlled conditions, in vitro neuronal cultures have become valuable experimental models given their controllability and ease of manipulation [19,20], particularly when combined with microfluidic devices [21,22]. While such systems have been used to study tau seeding and spreading [23], most experiments have not examined network-wide changes, such as alterations in the spatiotemporal structure of activity or disruptions of functional connectivity. In the present study, mouse primary neuronal cultures were exposed to either wild-type tau or seed-competent pTau and were monitored for sixteen days using GCaMP6s-based calcium imaging, with a comparison of dynamic traits and functional organization at day 12 in vitro (DIV). Despite exploring different pTau treatments and protocols, we observed no clear signs of network degeneration following pTau exposure, detecting only a modest increase in overall network activity.

## 2. Materials and Methods

The procedures for preparing neuronal cultures and the various pathological tau forms are detailed below. Table 1 and Table 2 summarize the reagents, resources, equipment, and software used for experiment preparation, data acquisition, and analysis.

### 2.1. Preparation of Sarkosyl-Insoluble Fractions from Mice’ Brains

P301S mice are a transgenic model genetically engineered to overexpress the shortest human tau isoform (0N4R) carrying the P301S mutation. This mutation is associated with an inherited form of frontotemporal dementia (FTD) [24].

Briefly, frozen brains were weighed and homogenized by using a Dounce homogenizer in a proportion of 1:10 of fresh, ice-cold homogenization buffer [0.8 M NaCl, 1 mM EGTA, 10% sucrose, 0.01 M Na_2_H_2_P_2_O_7_, 0.1 M NaF, 2 mM Na_3_VO_4_, 0.025 M β-glycerolphosphate, 0.01 M Tris-HCl, pH 7.4] supplemented with protease inhibitors (Hoffmann-La Roche, Basel, Switzerland). After centrifugation at 16,000 rpm for 22 min at 4 °C, the resulting supernatant (SN1) was collected. The pellet was then resuspended in a proportion of 1:5 with homogenization buffer and centrifuged again at 14,000 rpm for 22 min at 4 °C. The resulting supernatant (SN2) was combined with SN1, and the mixture (SN1 + SN2) was incubated with 0.1% sarkosyl (Sigma-Aldrich, St. Louis, MO, USA) on a rotating shaker at room temperature for 1 h. The mixture was centrifuged at 35,000 rpm for 63 min at 4 °C. The supernatant was discarded, and the remaining pellet (sarkosyl-insoluble fraction) was resuspended in 50 mM Tris-HCl, pH 7.4 (200μL/g starting material), aliquoted and stored at −80 °C until use. Protein concentrations were determined using the Pierce^TM^ BCA assay kit (Sigma-Aldrich), and equal amounts of protein were analyzed by immunoblot.

#### 2.1.1. Dodecyl Sulfate-Polyacrylamide Gel Electrophoresis

Samples were characterized through dodecyl sulfate-polyacrylamide gel electrophoresis (SDS-PAGE). Sarkosyl-insoluble fractions were mixed with 2× Laemmli sample buffer (Bio-Rad) and denatured for 10 min at 100 °C. The obtained solutions were then loaded on 10% SDS-PAGE gels and electrophoresed using a constant voltage of 60 V for 15 min, followed by 100 V for 1 h.

#### 2.1.2. Western Blot

The proteins were transferred onto nitrocellulose membranes through electrophoretic transfer, run at 100 V for 1 h. Following transfer, the membranes were blocked with 5% fat-free milk in Tris-buffered saline containing 0.1% Tween 20 (TTBS, Sigma-Aldrich, Madrid, Spain) for 1 h at room temperature. Membranes were incubated overnight with a buffer containing TTBS, 0.02% azide and the primary antibody mix [mouse anti-4R-tau (1:1000) (Merck Millipore, Burlington, MA, USA), mouse anti-β-Tubulin (1:10,000) (BioLegend, San Diego, CA, USA)], at 4 °C on a shaker. After that, membranes were washed three times with TTBS and incubated with the appropriate secondary antibody conjugated with horseradish peroxidase, which was diluted 1:1000 in 5% fat-free milk in TTBS for 1 h at room temperature. Subsequently, the membranes were rinsed three times with TTBS for 10 min each. Finally, membranes were revealed with the ECL-plus chemiluminescence western blot kit (Amersham-GE Healthcare, Amersham, UK).

### 2.2. Preparation of Primary Neuronal Cultures from Embryonic Cortical Mice

Tissue manipulation and neuronal culture procedures were conducted in accordance with the regulations of the Ethical Committee for Animal Experimentation of the University of Barcelona (approved ethical order B-RP-094/15-7125 of 10 July 2015) and the laws for animal experimentation of the Generalitat de Catalunya (Catalonia, Spain).

**Figure 1:**
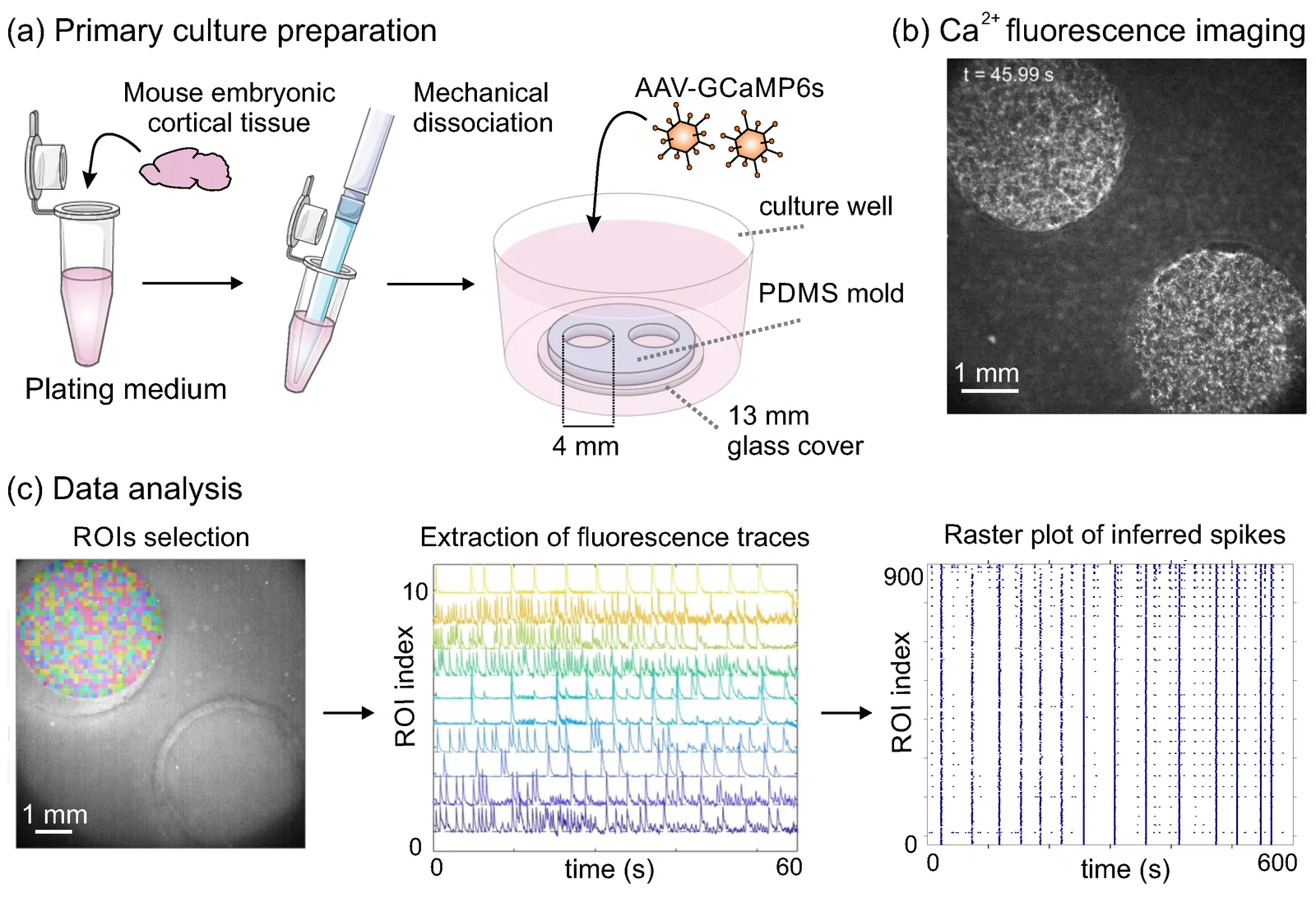
Culture preparation, monitoring and data analysis. (**a**) Culture preparation. Cortical tissue from mouse embryos was mechanically dissociated and plated onto twin 4 mm diameter PDMS cavities over glass. At DIV 1 cells were transduced with the calcium fluorescent indicator GCaMP6s. (**b**) Representative image of the twin cultures during a fluorescence calcium imaging recording. Bright areas indicate active neurons. (**c**) Data analysis pipeline using the software Netcal. A circular grid of 30×30 regions of interest (ROIs) was defined for each cavity, and the average fluorescence trace of each ROI as a function of time was extracted. Colored squares on the image identify the ROIs. Each trace, colored for clarity to separate different ROIs, was subsequently analyzed to detect sharp fluorescence peaks, which were ascribed as neuronal activations, producing raster plots that depicted the collective dynamics of the cultures. Blue dots represent neuronal activity, and apparent vertical lines indicate network bursts. Panels (**a**,**b**) of the figure used images from Servier Medical Art, licensed under Creative Commons Attribution 3.0 Unported License.

Prior to cell culturing, 13 mm coverslips (#1 Marienfeld-Superior, Lauda-Königshofen, Germany) were cleaned in 70% nitric acid for 90 min, rinsed twice in double-distilled water and then sonicated in a 96% ethanol bath for 20 min. Coverslips were flame-dried and attached to Polydimethylsiloxane molds (PDMS, Dow Corning, Midland, MI, USA) made of a 1 mm thick PDMS layer 13 mm in diameter that contained two circular cavities 4 mm in diameter each. The PDMS bonded strongly to the glass coverslips during subsequent autoclaving, effectively forming twin PDMS cavities on a glass substrate (Figure 1). The PDMS molds contained two 4 mm wells to allow their simultaneous recording, thereby doubling statistical yield from a single recording. The glass-PDMS assemblies were placed into 4-well plates (Nunc, ThermoFisher Scientific, Waltham, MA, USA) and treated overnight with a 10 mg/mL Poly-L-Lysine (PLL, Sigma-Aldrich) to promote cell attachment and ensure a homogeneous distribution of neurons on the glass.

Cortical tissue from CD1 mouse embryos at day 16 of development (E 16) was extracted, mechanically dissociated by repeated pipetting and plated in the PDMS-glass systems (Figure 1a). Mice were provided by Charles River Laboratories (Saint Germain Nuelles, France). Dissections were performed in L-15 medium (Gibco, ThermoFisher Scientific) previously enriched with 4% glucose 1 M, 1% glutamax (Sigma-Aldrich) and 0.4% gentamicin (Sigma-Aldrich). Dissociated neurons initially developed on ‘plating medium’ [90% Eagle’s MEM-enriched with 0.6% glucose, 1% 100X glutamax, and 20μg/mL gentamicin, with 5% horse serum, 5% fetal calf serum and 1μL/mL B27]. Isolated neurons were plated with a final concentration of $10^{6}$ cells/mL.

At day in vitro (DIV) 1, the genetically encoded fluorescent calcium indicator GCaMP6s (AAV9.Syn.GCaMP6s.WPRE.SV40, Viral Vector Production Unit, Barcelona, Spain) was introduced into the cell culture via viral transduction using adeno-associated viruses (AAVs), which expressed the indicator under the synapsin-I promoter of mature neurons, as illustrated in Figure 1a. At DIV 5, plating medium was switched to ‘changing medium’ [90% Eagle’s MEM-enriched with 0.6% glucose, 1% 100X glutamax, and 20μg/mL gentamicin, with 10% horse serum and 0.5% FUDR] to stop glial cell proliferation. Medium was then replaced at DIV 7 to ‘final medium’ [Enriched 90% Eagle’s MEM with a 10% horse serum] and refreshed every two days. Cultures were incubated at 37 °C, 5% CO_2_ and 95% humidity.

The prepared cultures contained both neurons and glial cells, but only neuronal activity was monitored through the GCaMP6s indicator, as described later. All cultures consisted of approximately 80% excitatory neurons and 20% inhibitory ones [25].

#### 2.2.1. Emulation of Tauopathies In Vitro I: Sarkosyl-Insoluble Fraction of P301S Mice

**Figure 2:**
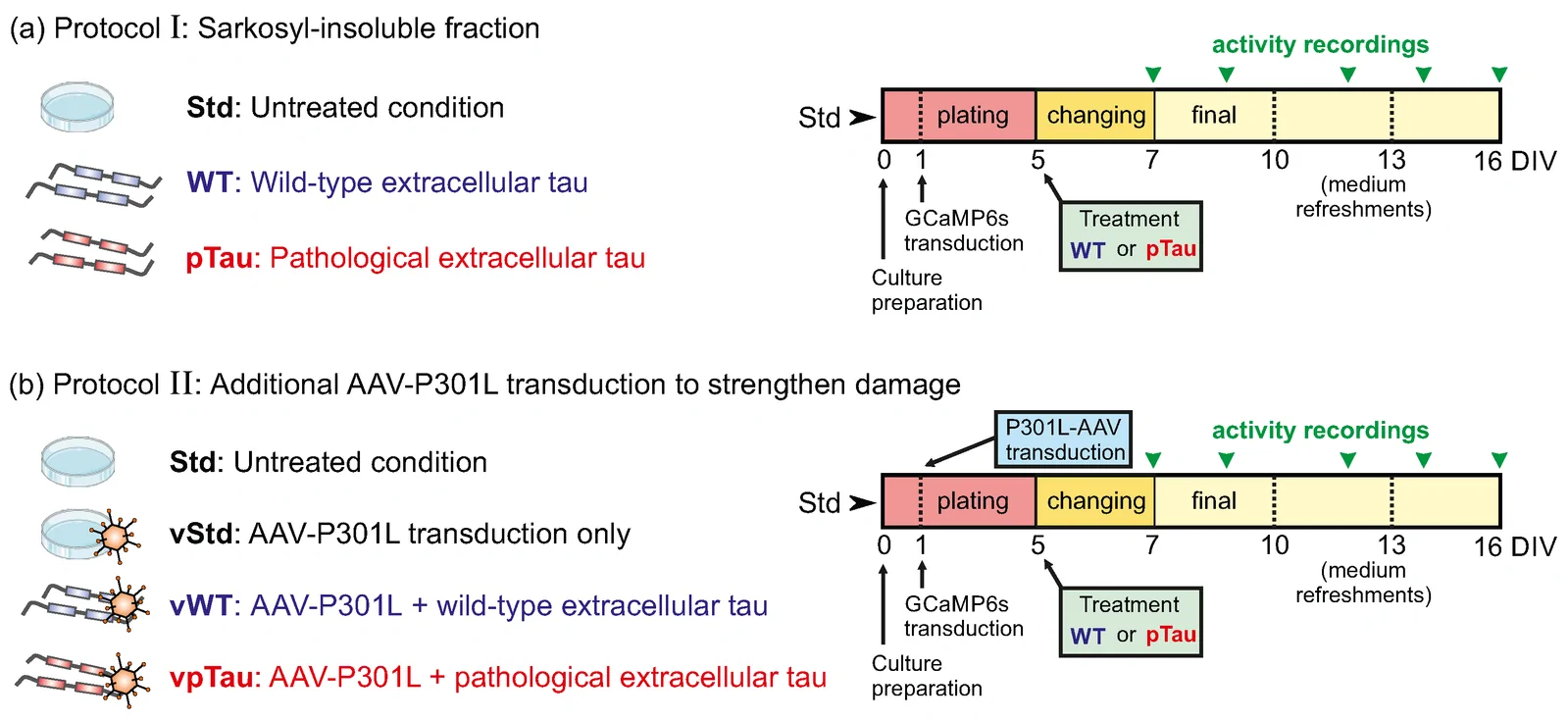
Summary of protocols used to induce pTau-related damage and corresponding diagrams for the application of treatments and culture maintenance.The diagrams indicate key days in vitro for culture manipulation, as well as the characteristic time points for recording spontaneous activity via calcium fluorescence imaging (green arrowheads). (**a**) Protocol I, based on the sarkosyl-insoluble fraction derived from P301S cells. (**b**) Protocol II, which incorporates AAV-P301L transduction to enhance the damaging action of pTau. Figure panels utilize images from Servier Medical Art, licensed under Creative Commons Attribution 3.0 Unported License.

In a first group of experiments, a potential pathological state was induced in the neuronal cultures through the administration of extracellular tau, obtained from the sarkosyl-insoluble brain fraction of P301S mice, and the resulting network behavior was compared with that of the corresponding wild-type controls. Treatments were administered at DIV 5, when the culture medium was switched from ‘plating’ to ‘changing’. The experimental conditions, summarized in Figure 2a, were as follows:

- Standard culture (Std): Neurons developed following the standard laboratory protocol described above. These measurements provided a reference for normal network development and activity that could be compared with treated cultures.
- Wild-type extracellular tau (WT): Medium containing P301S (−/−) sarkosyl-insoluble fraction, which was hypothesized to be innocuous, was administered to the cultures.
- Pathological extracellular tau (pTau): Medium containing potentially harmful tau, derived from P301S (+/−) sarkosyl-insoluble fraction, was administered to the cultures.

Neuronal cultures were prepared in sets of four using appropriate culture plates, ensuring that all cultures had the same cell density and originated from the same dissection. Within each set, one culture (containing two wells) was assigned to the Std condition, one to the WT condition, and two to the pTau condition. This strategy was designed to foresee possible degeneration and compromised development in pTau-treated cultures.

The treatments were administered only once and were not refreshed during subsequent media changes. This decision was based on evidence showing that tau protein is internalized by cells within 24 h of exposure [22,26]. Accordingly, a single application of tau is sufficient for cellular uptake, making repeated treatments unnecessary. By administering tau only once and allowing approximately three days before replacing the culture medium, we ensured that cells had an adequate time window to internalize the protein. Treatment concentrations were evaluated and adjusted to 10 μg/mL of total protein. A total of 28 wells (14 cultures) from approximately 8 different dissections were finally considered. Of these, 8 were assigned to the Std condition, 8 to WT, and 12 to pTau. Subsequent analyses of network dynamic descriptors (see Section 2.4.3) occasionally resulted in insufficient data points for reliable quantification. For this reason, some WT plots contain 7 data points instead of 8.

#### 2.2.2. Emulation of Tauopathies In Vitro II: P301L Mutated Tau Delivered Through AAVs

In a second group of experiments, potential damage was accelerated by transducing the cultures at DIV 1 with $2\times 10^{7}$ viral particles encoding full-length human tau with the P301L mutation (AAV-P301L) expressed under the synapsin-I promoter. At DIV 5, cultures were then treated with the sarkosyl-insoluble brain fraction of P301S mice (WT or pTau) coinciding with medium change, as above. As summarized in Figure 2b, the experimental conditions in this group were:

- Standard culture (Std): Neurons developed following the standard laboratory protocol.
- Viral P301L condition (vStd): Cells were infected at DIV 1 with AAV-P301L to induce expression of P301L-mutated tau. From this time point onwards, cultures were manipulated as in the standard condition.
- Viral P301L + wild-type tau (vWT): Cells were infected with AAV-P301L at DIV 1. At DIV 5, P301S (−/−) sarkosyl-insoluble fraction (WT) was added.
- Viral P301L + pTau (vpTau): Cells were infected with the AAV-P301L at DIV 1. At DIV 5 P301S (+/−) sarkosyl-insoluble fraction (pTau) was added.

Neuronal cultures were prepared in sets of four using appropriate culture plates, ensuring again that all cultures had the same cell density and originated from the same dissection. Within each set, each culture (two wells) was assigned to a distinct experimental condition. As in protocol I, all treatments with P301S sarkosyl-insoluble fraction were administered only once at a concentration of 10 μg/mL. A total of 30 wells (15 cultures) from approximately 4 different dissections were considered. Of these, 6 were assigned to the Std condition, 8 to vStd, 5 to vWT, and 11 to vpTau.

### 2.3. Toxicity Analysis

#### 2.3.1. Tau Treatments

To study the cytotoxic effects of extracellular seed-competent tau, primary neuronal cell cultures were treated with sarkosyl-insoluble fractions from P301S mice at 6 DIV for 48 h. For treatments with sarkosyl-insoluble fractions from P301S (+/−) mice, 20 μg of total protein was diluted in 500 μL of culture medium. Likewise, primary neurons transduced with AAV-P301L were either left unperturbed or incubated with 20 μg of total protein from sarkosyl-insoluble fractions derived from P301S (+/−) mice, diluted in 500 μL of culture medium. Altogether, these treatments provided three possible toxicity conditions. Untreated cells were used as negative controls. As a positive control for cell death, cultures were exposed to 70% ethanol (EtOH) for 15 min. Thus, five conditions were explored.

For cytotoxicity experiments, cells were plated in quadruplicate for each condition to compare 4 different days (2, 5, 7 and 10 days) of treatment. For each condition and day, at least 8 cultures were prepared for analysis and statistical quantification.

#### 2.3.2. AlamarBlue^®^ Cell Viability Assay

Tau-related cytotoxicity was assessed by measuring cellular metabolic integrity in primary neuronal cultures at 2, 5, 7, and 10 days of treatment (DOT) using the alamarBlue Cell Viability Reagent (Thermo Fisher Scientific, Waltham, MA, USA), according to the manufacturer’s instructions. Briefly, on the day of the assay, positive controls for cell death were prepared by replacing the culture medium with 300 μL of 70% ethanol for 15 min. Subsequently, the media of all wells (i.e., tau-treated and control wells) were aspirated and replaced with freshly prepared culture medium.

AlamarBlue reagent was then added directly to each well at a final concentration of 10% (*v*/*v*), and plates were incubated at 37 °C with 5% CO_2_ for 4 h. For fluorescence measurements, 100 μL of medium from each replicate was transferred in triplicate to a 96-well black-bottom plate (Costar, Tewksbury, MA, USA). Fluorescence intensity was measured using an Infinite M200 PRO microplate reader (Tecan, Männedorf, Switzerland) with excitation at 530 nm and emission at 570 nm.

Fluorescence values, reflecting the reduction of alamarBlue, were normalized to untreated controls, which were set to 100% for each independent experiment. At 10 DOT, alamarBlue measurements were complemented by analyses of (i) the total number of cells, determined by Hoechst staining, and (ii) the proportion of neurons, calculated as the ratio of NeuN-positive cells to the total number of Hoechst-positive nuclei.

#### 2.3.3. Immunocytochemistry

For standard fixation, cultured primary neurons were washed once with pre-warmed 0.1 M PBS and fixed in 4% paraformaldehyde (PFA) for 15 min at room temperature. For removal of soluble proteins, cells were washed once with pre-warmed 0.1 M PBS and fixed in pre-chilled methanol for 15 min at −20 °C. Following fixation, cells were permeabilized and blocked for 1 h in blocking solution consisting of 5% normal goat serum (NGS), 0.1% Triton X-100, and 0.015 g/mL glycine in 0.1 M PBS containing 2% BSA.

Cells were then incubated overnight at 4 °C with primary antibodies diluted in antibody solution (2% NGS, 0.1% Triton X-100 in 0.1 M PBS). The following primary antibodies were used: mouse anti-human tau HT7 (1:600; Thermo Fisher Scientific, catalog no. MN1000), rabbit anti-GFP (1:500; Thermo Fisher Scientific, Waltham, MA, USA, catalog no. A11122), and mouse anti-NeuN (1:500; Merck Millipore, catalog no. MAB377). Appropriate Alexa Fluor 488 or Alexa Fluor 568 secondary antibodies (Thermo Fisher Scientific, Waltham, MA, USA) were diluted 1:500 in antibody solution and incubated for 2 h at room temperature. Cells were subsequently washed three times in 0.1 M PBS. Nuclei were counterstained with 1 μg/mL Hoechst 33342 (Invitrogen, Carlsbad, CA, USA) for 10 min at room temperature.

#### 2.3.4. Imaging

Fluorescence imaging was performed using an inverted Olympus IX71 microscope equipped with a high-speed Hamamatsu Orca Flash 4.0 camera and an LED light source (CoolLED pE-300white, Delta Optics, Madrid, Spain). Images were acquired using Olympus cellSens^TM^ software (v3.2.1, Olympus Corporation, Tokyo, Japan) and saved as 8-bit .tiff files.

#### 2.3.5. Image Processing and Data Acquisition

To quantify neuronal survival, the proportion of neurons was calculated as the ratio of NeuN-positive cells to total Hoechst-positive nuclei. Twelve replicates per condition were analyzed. For each replicate, 15 randomly selected fields per well were imaged at 20X magnification (2048×2048 pixels) using red (NeuN) and blue (Hoechst) channels. The two channels were subsequently combined for analysis. Identical exposure settings were applied across all experiments to ensure consistency.

Image analysis was performed using CellProfiler v4.2.1. A customized pipeline was developed to automatically identify and quantify Hoechst-positive nuclei and NeuN-positive neurons in each field.

### 2.4. Spontaneous Activity Data Acquisition and Analysis

#### 2.4.1. Calcium Fluorescence Imaging

Spontaneous activity in the designed conditions was monitored every two days over a 2-week period. Recordings commenced at DIV 7, when the fluorescence calcium signal was sufficiently strong, and ended at DIV 16, when cultures in standard conditions degraded and detached from the glass. For clarity in the presentation of results, detailed analyses of culture behavior were restricted to DIV 12, with overall developmental trends reported in the Supporting Information. Activity was recorded on a Zeiss Axiovert C25 inverted microscope equipped with a calcium imaging setup and a high-speed CMOS camera (Hamamatsu Orca Flash 4.1) that provided an image size of 1024×1024 pixels and a 8-bit gray scale levels. Two 4 mm diameter neuronal networks grown in the PDMS cavities were recorded simultaneously by using a 2.5X objective combined with an optical zoom (Figure 1b), providing a spatial resolution of 6.8μm/pixel. Recordings were carried out for 15 min at 33 frames per second.

#### 2.4.2. Data Analysis and Event Detection

Calcium imaging recordings were analyzed with the software Netcal [27] run in Matlab. For analysis, a set of 700 Regions of Interest (ROIs) were first set on each of the twin 4 mm wells, shaping a circular grid centered at the well (Figure 1c, left). Each ROI had a typical size of 14×14μm and contained about 1–3 neurons. The fluorescence intensity $F_{i}$(t) of each ROI *i* along the 15 min recording was next extracted, corrected from drifts and normalized as ${\left(\Delta F/F\right)}_{i}\;\left(t\right)\equiv \left(F_{i}\left(t\right)-F_{i,0}\right)/F_{i,0}$, where $F_{i,0}$ is the fluorescence level of ROI *i* at rest (Figure 1c, center).

The fluorescence trace of each ROI was converted into a time series representing neuronal activity using the Schmitt-trigger method [28]. This approach identifies an activity event when the fluorescence signal remains above an upper threshold for at least 100 ms and subsequently falls below a lower threshold. For most experiments, the thresholds were fixed at ${\mathrm{th}}_{\mathrm{high}}=1.5$ and ${\mathrm{th}}_{\mathrm{low}}=1.0$ (ΔF/F units) above the average noise level. However, in experiments exhibiting particularly low fluorescence amplitudes, these thresholds were lowered to ensure sufficient event statistics for the detection of network bursts and the estimation of front propagation velocities. The resulting train of detected events (‘1’ for the presence of activity and ‘0’ for its absence) for each ROI in the culture was presented as a raster plot (Figure 1c, right), which provided the main dataset for subsequent dynamical and effective connectivity analyses.

#### 2.4.3. Networks Dynamical Characteristics

The collective behavior of neuronal cultures was visualized using raster plots and was quantified through a set of descriptors linked to population-level dynamics. These descriptors were successfully applied in studies of neuronal cultures to quantify network alterations, e.g., in response to changes in connectivity [29] or in the context of disease-induced degeneration [30,31]. These descriptors enabled us to assess whether possible alterations induced by pathological tau translated into changes in network dynamics, and included ‘population activity’, which reflected the tendency of neurons to activate in highly coordinated events such as network bursts; the ‘velocity of propagation fronts’, which served as an indirect indicator of changes in network connectivity; and the ‘inter-burst interval’, which captured shifts in the frequency of network bursting.

(i) *Population activity*: Denoted as PA, it quantified the tendency for the neurons in the network to activate together in a short time window. It ranged from 0 (no collective activity) to 1 (full network activation). The latter conceptually corresponded to vertical bands of activity in the raster plots. To calculate PA, a 1 s sliding window scanned the raster plot in 0.1 s steps and counted the number of independent ROIs in the network that activated within the window. The resulting number was then divided by the total number of ROIs to obtain the fraction of the network that activated together. Sharp peaks in PA indicated strongly coordinated activity and were referred to as *network bursts*. These bursts were considered significant whenever their amplitude, denoted as PA_*b*_, satisfied the condition ${\mathrm{PA}}_{b}>\mu_{\mathrm{bgnd}}+3\cdot SD_{\mathrm{bgnd}}$, where $\mu_{\mathrm{bgnd}}$ and $SD_{\mathrm{bgnd}}$ represented the mean and standard deviation of the background activity, respectively. Typically, significant bursts were those with ${\mathrm{PA}}_{b}>0.1$, i.e., 10% of the network. All significant burst amplitudes ${\mathrm{PA}}_{b}$ observed in a recording were averaged to provide the culture’s characteristic PA value, and pooled together with other experimental replicates.

(ii) *Propagation velocity of network bursts*: Denoted by *v*, this metric quantified the speed at which activity propagated through the network, ultimately reflecting the network’s connectivity. The velocity was determined using previous findings showing that neuronal cultures grown on glass exhibit network bursts that propagate as circular fronts originating from a point $\left(x_{0},y_{0}\right)$ referred to as the ‘burst initiation point’ [29,32]. The velocity was estimated by plotting the Euclidean distance $D_{i}$ of each ROI *i* from the initiation point, $D_{i}={\left[{\left(x_{i}-x_{0}\right)}^{2}+{\left(y_{i}-y_{0}\right)}^{2}\right]}^{1/2}$, as a function of the time of activation $t_{i}$ of that ROI. The slope of the resulting linear fit D(t) provided the average propagation velocity. Only fits with a correlation coefficient above 0.7 were considered valid. All propagation velocities observed in a neuronal network were averaged to obtain the culture’s characteristic propagation velocity value.

(iii) *Inter-burst interval*: Denoted as IBI, it was defined for each studied network as the average time elapsed between two consecutive network bursts, and was calculated as $\mathrm{IBI}={\langle T_{k+1}-T_{k}\rangle }_{k}$, where $T_{k}$ is the activation time of each burst *k*.

Only neuronal cultures exhibiting at least five network bursting events were included in the analysis of PA, velocity, and IBI; otherwise, they were excluded. For each analyzed independent culture, the mean value of each descriptor was used for statistical comparisons across replicates and experimental conditions. Because some cultures were excluded from analysis of dynamical characteristics, the number of independent replicates contributing to the box plots in the Section 3 may vary between figures.

#### 2.4.4. Effective Connectivity Analysis and Network Metrics

Because network-wide structural connectivity in neuronal cultures cannot be directly accessed, nor its changes following perturbations monitored in detail, we relied on effective connectivity as a proxy for structural connectivity [33]. Effective connectivity captures causal interactions between neurons inferred from their activity patterns. The resulting connectivity matrix can be analyzed to extract a set of network metrics that quantify network-level communication among neurons, and that may change following degeneration-induced damage, as shown in previous in vitro studies [30,31]. These metrics include ‘global efficiency’ ($G_{E}$), which quantifies the ability of neurons to exchange information across the network, with higher $G_{E}$ values indicating a more strongly integrated network; and the ‘community statistic’ (*Q*), which measures the tendency of neurons to organize into smaller groups or communities rather than interacting as a single unified network, with higher *Q* values reflecting greater network fragmentation.

Causal relationships between pairs of ROI spike trains were obtained using a modified version of Generalized Transfer Entropy (GTE) implemented in Matlab, as described in [33,34,35]. To carry out the analysis, binarized vectors of neuronal activity trains for each ROI were built, with each vector containing the 15 min recording in 20 ms bins whose values were either 1 (presence of an activity event) or 0 (absence of activity). An effective connection from ROI *I* to ROI *J* was inferred whenever the information contained in *I* significantly improved the prediction of future states of *J*. Instant feedback was present and Markov order was set to 2 [33]. The significance of the inferred connections was established by comparing the transfer entropy estimate $TE_{I\to J}$ with the joint distribution of all input *X* to *J* and output *I* to *Y* (for any *X* and *Y*), as

(1) $$z_{IJ}=\frac{TE_{I\to J}-\langle TE_{\mathrm{joint}}\rangle }{\sigma_{\mathrm{joint}}},$$

where $\langle TE_{\mathrm{joint}}\rangle$ and $\sigma_{\mathrm{joint}}$ are the average value of the joint distribution and its standard deviation, respectively. A connection was then deemed significant for $z_{IJ}\geq 2$, setting the corresponding effective connection to ‘1’ (presence of an effective link); otherwise the connection was set to ‘0’ (absence of an effective link). The threshold value of 2 was considered optimal to capture both strong neuron-to-neuron interactions and whole network communication [29,34].

The final effective connectivity networks were directed but binary, and were stored in an adjacency matrix $A=\{a_{IJ}\}$ for the computation of the following network metrics using the Brain Connectivity Toolbox [36] run in Matlab.

(i) *Global efficiency $G_{E}$* It captured the capacity of the network to exchange information as a whole [37], and was defined as

(2) $$\begin{array}{c} G_{E}=\frac{1}{N(N-1)}\sum_{0\leq i,j\leq N}\frac{1}{d(i,j)}, \end{array}$$

where *N* is the number of ROIs and d(i,j) the shortest topological path connecting ROIs *i* and *j*, with non-connected ROIs procuring d(i,j)=∞. $G_{E}$ varied between 0 and 1. A value of $G_{E}=0$ indicates that the network was an ensemble of disconnected ROIs, while $G_{E}=1$ indicates that any neuron immediately communicates with any other, i.e., information was swiftly exchanged at a global scale.

(ii) *Community statistic Q*: It quantified the tendency of neurons to form functional communities, i.e., groups of neurons that were more connected within their group than with the rest of the network. *Q* was defined as [38]

(3) $$\begin{array}{c} Q=\frac{1}{2m}\sum_{0\leq i,j\leq N}\left(a_{ij}-\frac{k_{i}k_{j}}{2m}\right)\delta \left(c_{i},c_{j}\right),\;\;m=\frac{1}{2}\sum_{i,j=1}^{N}a_{ij}, \end{array}$$

where *N* is the number of ROIs, $a_{ij}$ is the (i,j) element of the effective connectivity matrix A, $k_{i}=\sum_{j=1}^{N}a_{ij}$ is the sum of the links attached to neuron *i*, $c_{i}$ denotes the community assignment of node *i*, and δ(u,v) is the Kronecker Delta with δ(u,v)=1 for u=v and 0 otherwise. Optimal community structure was identified using the Louvain algorithm [38]. *Q* varies between 0 and 1, where Q≃0 indicates the absence of communities (the entire network behaves as a single community), whereas a value Q≃1 indicates that each ROI forms an isolated community in a fully disconnected network. Values of Q≳0.3 typically indicate the presence of well-defined communities in the network [29].

Network metrics were calculated independently for each 4 mm network and data pooled together across replicates and experimental conditions for statistical comparison. For cases in which a representative connectivity matrix is shown in the Section 3, one matrix of the two 4 mm networks was selected at random.

### 2.5. Statistical Analysis

Quantitative data were analyzed using one-way analysis of variance (ANOVA) followed by Tukey’s post-hoc test for multiple group comparisons. Statistical significance was defined as p<0.05. Effect sizes were quantified using eta-squared ($\eta^{2}$), which represents the proportion of total variance explained by the experimental factor. Larger $\eta^{2}$ values indicate a greater contribution of the experimental condition to the observed variability. According to conventional criteria, $\eta^{2}$ values of 0.01, 0.06, and 0.14 correspond to small, medium, and large effects, respectively. Data were presented as mean ± standard deviation (SD). Statistical analyses and graph generation were performed using Origin 9.1 (OriginLab Corporation, Northampton, MA, USA) and MATLAB (MathWorks Inc., Natick, MA, USA). Significance levels are indicated as * *p* < 0.05, ** *p* < 0.01; *** *p* < 0.001. Non-statistically significant differences are omitted from the graphs for clarity of presentation.

## 3. Results

Our experimental study combined a detailed characterization of neuronal dynamics and functional connectivity at DIV 12 with a longitudinal analysis spanning DIV 7 to DIV 16. DIV 12 was selected as the primary time point because neuronal networks had reached a sufficient degree of maturation, while immunostaining experiments confirmed the presence of seed-competent tau species within the cultures. Consequently, DIV 12 represented the most appropriate stage to compare dynamic and connectivity descriptors with the underlying pathological state. In the following. we first present the results of pathological tau characterization and neuronal toxicity, followed by the analysis of network dynamics and connectivity at DIV 12. Finally, we describe the developmental trajectories revealed by the longitudinal analysis.

### 3.1. Sarkosyl-Insoluble Fractions from P301S Mouse Brains Contain Pathological Tau

The tau biosensor cell line has served as a reliable source to investigate the presence of proteopathic seeding activity—abnormal protein folding, aggregation, or accumulation—in biological specimens since its development in M. I. Diamond’s laboratory in 2014 [39] and later incorporated in other studies [40]. This engineered cell line expresses a fusion of the tau repeated domain with the disease-associated P301S mutation, coupled to either the cyan or yellow fluorescence proteins. The addition of extracellular seed-competent tau increases the recruitment of fragments of this protein present in the biosensor cell line, leading to the formation of circular-like intracellular inclusions that can be quantified via flow cytometry or fluorescence imaging.

**Figure 3:**
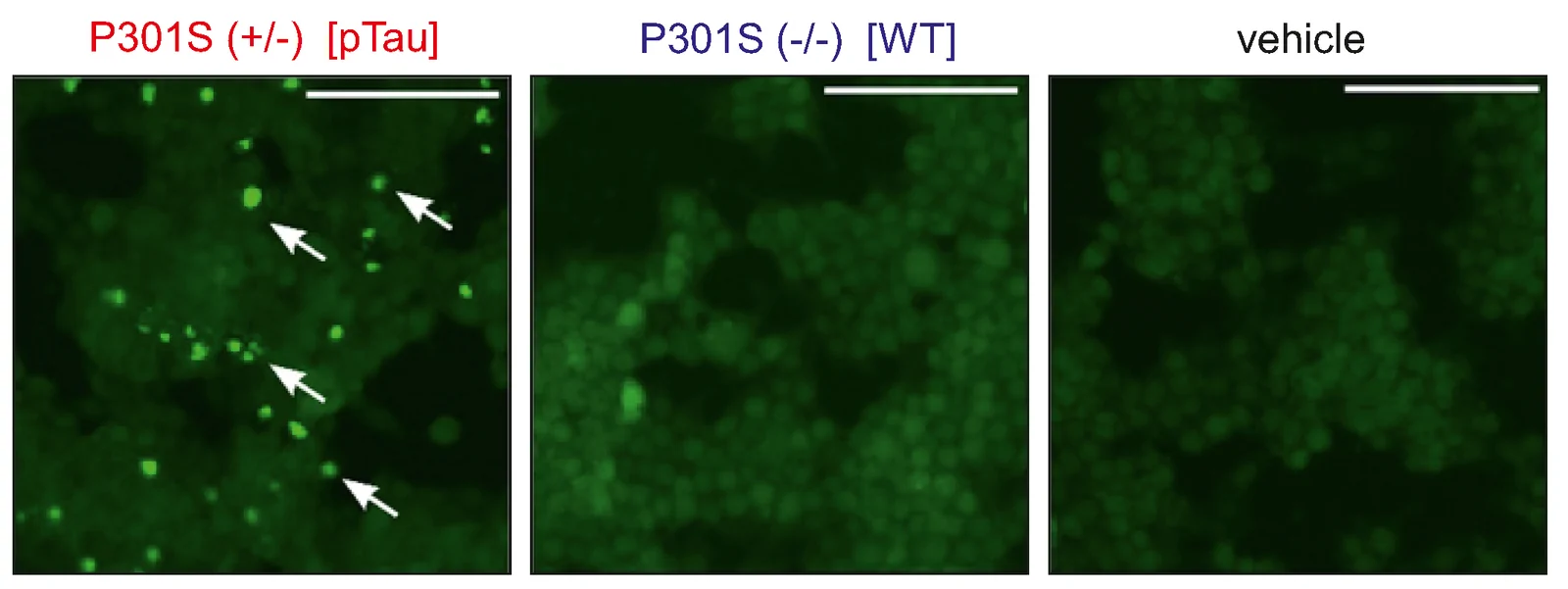
Characterization of the tau biosensor cell line. The images show in vitro tau biosensor cells at DIV 12 treated with sarkosyl-insoluble fractions derived from P301S (+/−) mice (pTau), their innocuous analogous P301S (−/−) (WT), as well as empty liposomes (vehicle), which correspond to sarkosyl-free controls. Only cells treated with P301S (+/−) show a widespread presence of fluorescent inclusions, visible as bright spots (white arrows) against the grainy culture background, indicating the presence of pTau. Scale bars are 50μm.

In this context, and to verify the presence of seed-competent tau species prior to their use in spontaneous activity recordings of primary neuronal cultures (protocol I), we examined in vitro biosensor cells at DIV 12 that were exposed to sarkosyl-insoluble fractions obtained from the brains of P301S (+/−) mice, which are considered to carry pTau. In parallel, P301S (−/−) sarkosyl-insoluble fractions were also analyzed to confirm the absence of pTau inclusions in the wild-type condition. As a control, we considered empty liposomes used as vehicles. As shown in Figure 3, only P301S (+/−) samples triggered the formation of fluorescent inclusions (highlighted by white arrows), whereas neither the P301S (−/−) samples nor the vehicle preparations produced detectable signals. These results confirmed that sarkosyl-insoluble fractions extracted from P301S (+/−) mouse brains contain pathological, seed-competent tau species.

### 3.2. Cortical Neurons in Culture Express P301L Human Tau

**Figure 4:**
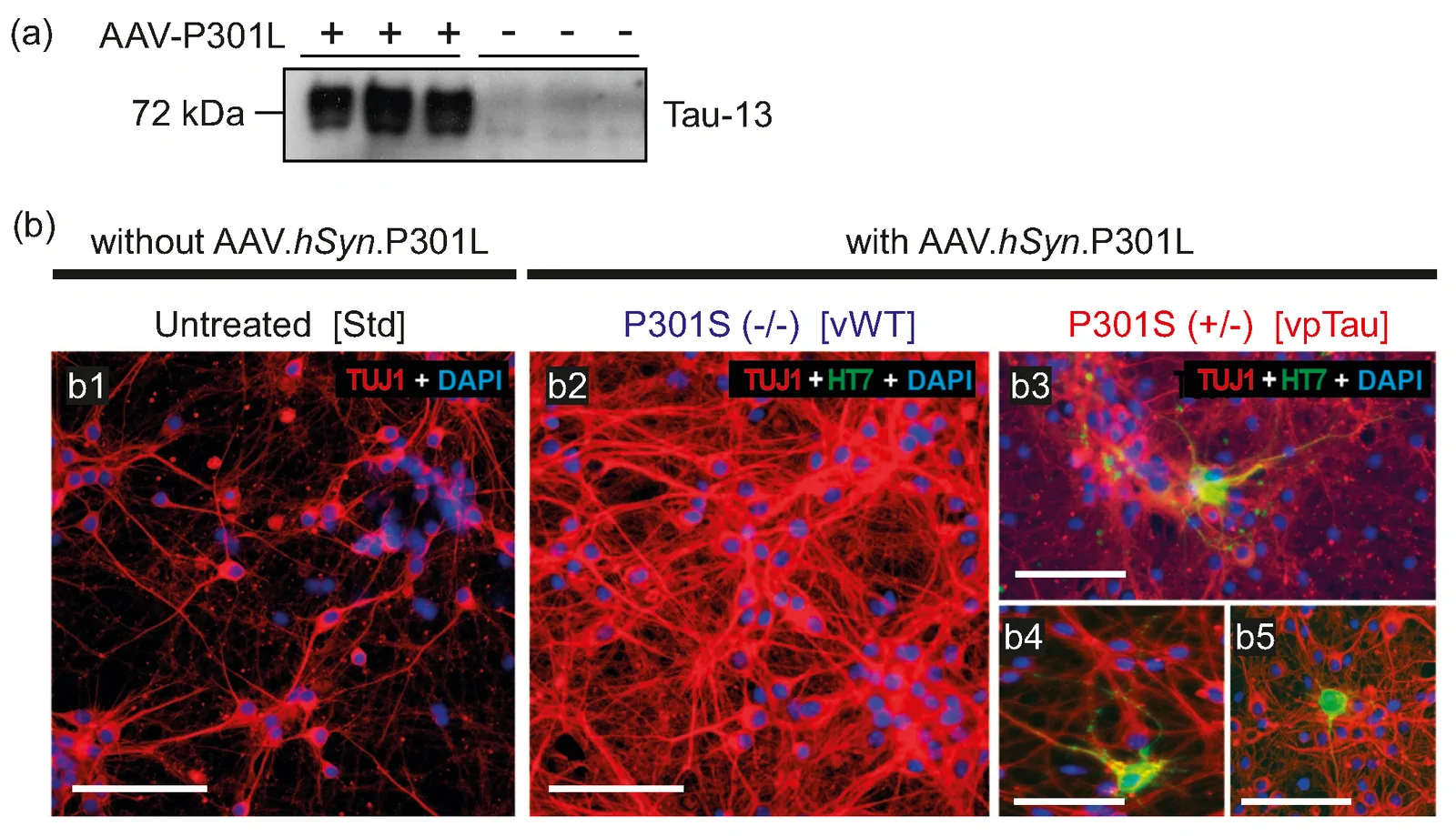
Expression of P301L human tau in mouse primary cortical neurons. (**a**) Immunoblot analysis at DIV 16 that confirms transgene specificity. Cortical lysates probed with tau-13 antibody showed human tau expression exclusively in the AAV-P301L-transduced cultures (‘+’ symbol), with no signal in control samples (‘−’ symbol). (**b1**) Representative immunostaining image of an untreated primary cortical culture (i.e., not infected with AAV-hSyn-P301L) at DIV 16, used as reference (Std condition). Neurons and their processes are TUJ-1 positive (red) and cell nuclei are DAPI positive (blue). (**b2**–**b5**) Cortical neurons infected with AAV-hSyn-P301L and subsequently treated with P301S (−/−) sarkosyl-insoluble extracts (vWT, panel (**b2**)) or with P301S (+/−) extracts (vpTau, panels (**b3**–**b5**)). Neurons were immunolabeled at DIV 16 with anti-HT7 antibody (green) to stain human tau, along with TUJ1 and DAPI. Green HT7 staining is observed only in panels (**b3**–**b5**), indicating neuronal uptake of pTau species [P301S (+/−)]. Scale bars are 100μm (panels (**b1**,**b2**)) or 50μm (**b3**–**b5**).

We verified by western blot (Figure 4a) that virally-transduced cells expressed human tau. We next evaluated the capacity of human P301L to form insoluble tau aggregates. For that, we followed protocol II described in Methods and prepared the four experimental conditions indicated. At DIV 16 (corresponding to the final stage of neuronal culture monitoring), cells were fixed, treated with methanol to remove soluble tau, and immunostained with the anti-HT7 antibody (green) to detect insoluble tau, together with Hoechst (blue) to label cell nuclei. As shown in Figure 4b, insoluble tau was absent in both untreated cultures (panel b1) and cultures exposed to P301S (−/−) (panel b2). In contrast, robust HT7-positive aggregates were observed in the P301S (+/−) pTau condition (panels b3–b5). We therefore confirmed that cultured cells not only expressed P301L human tau but were also capable of forming insoluble, pathological aggregates. Additional images taken in a larger field of view are provided in Supplementary Figure S1.

### 3.3. Toxicity Assays Show No Cellular Affectations Across Treatments

**Figure 5:**
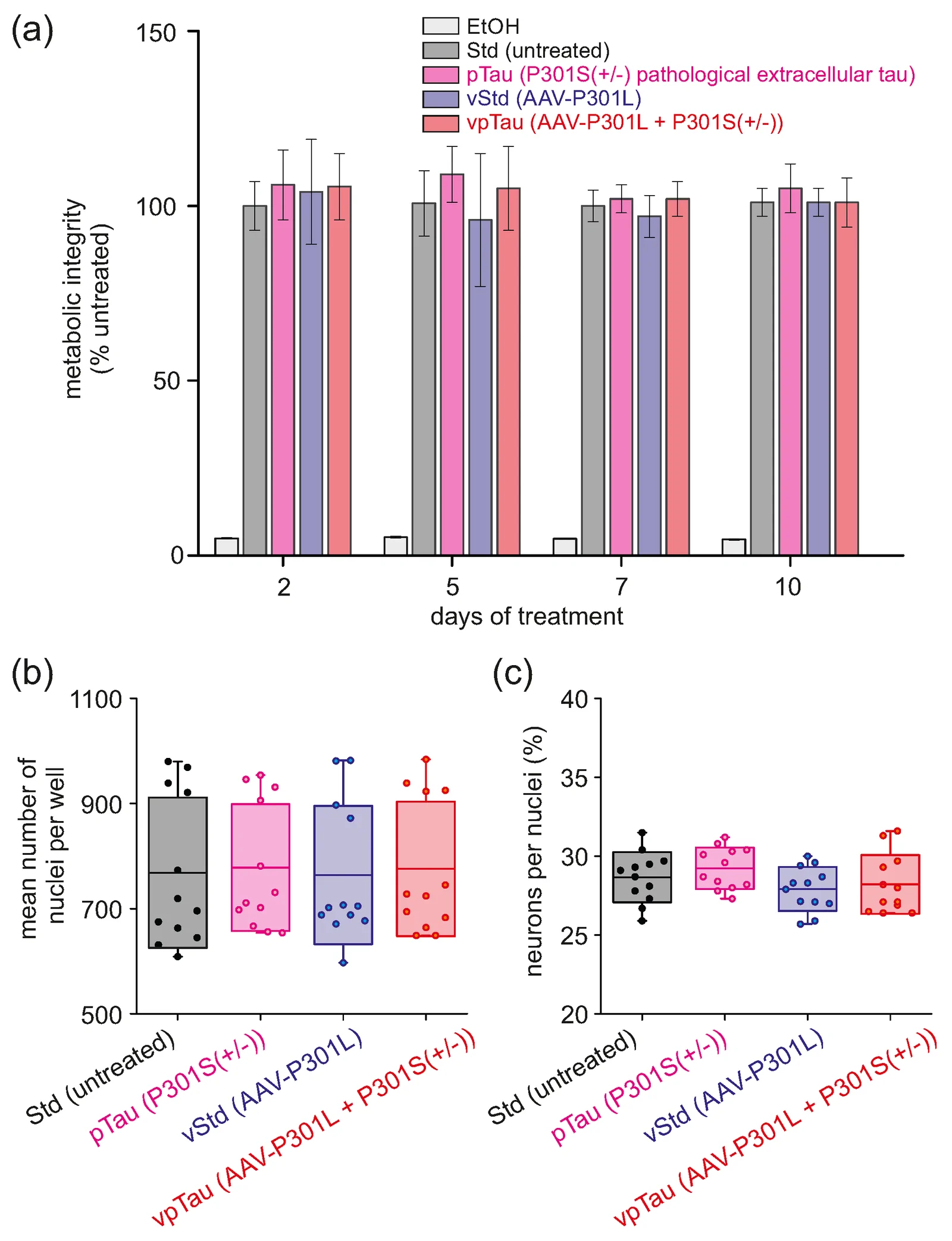
Extracellular seed-competent tau present in sarkosyl-insoluble fractions derived from P301S (+/−) mice is not toxic to primary cortical cultures overexpressing P301L tau. Primary neuronal cell cultures were left untreated, treated with sarkosyl-insoluble fractions from P301S (+/−) mice, transduced with AAV-P301L without further treatment, or transduced with AAV-P301L and subsequently treated with sarkosyl-insoluble fractions from P301S (+/−) mice after DIV 6. As a positive control for cell death, cultures were exposed to 70% ethanol (EtOH) for 15 min. (**a**) Time-course analysis of AlamarBlue^TM^ assays in primary neuronal cell cultures at 2, 5, 7, and 10 days of treatment (DOT). No differences in metabolic activity among experimental conditions were observed at any time point. Data are presented as mean ± SD from at least eight independent experiments. Statistical analysis was performed using one-way ANOVA followed by Tukey’s post-hoc test for group comparisons. All experimental groups differed significantly from the EtOH control, but this comparison is omitted from the graph for clarity. (**b**) Effects of each treatment on the total number of nuclei at 10 DOT. Each dot represents the average of 15 pictures from 12 independent experiments. Horizontal bars indicate the mean, colored boxes represent mean ± SD, and whiskers indicate minimum and maximum values. No significant differences were detected between conditions (one-way ANOVA with Tukey’s post-hoc test). (**c**) Neuronal viability at 10 DOT, expressed as the percentage of neurons (NeuN-positive cells) relative to the total number of cells (Hoechst-positive). Each dot represents the average of 15 images from 12 independent experiments. Horizontal bars indicate the mean, colored boxes represent mean ± SD, and whiskers indicate minimum and maximum values. No significant differences were detected between conditions (one-way ANOVA with Tukey’s post-hoc test).

We quantitatively addressed the putative impairment of cellular metabolism using the alamarBlue assay (Figure 5a, all ANOVAs providing p>0.05 with corresponding moderate-to-large effect sizes, $\eta_{\mathrm{DIV}2}^{2}=0.057$, $\eta_{\mathrm{DIV}5}^{2}=0.292$, $\eta_{\mathrm{DIV}7}^{2}=0.147$, $\eta_{\mathrm{DIV}10}^{2}=0.085$). No differences in viability were found in neurons over-expressing P301L tau treated with P301S (+/−) compared to non-transduced neurons, neither under P301S (+/−) treatment alone nor compared to the untreated control. Additionally, at 10 days of treatment, we did not detect significant differences in the total number of nuclei among groups (Figure 5b, one-way ANOVA with F(3,44)=0.03, p=0.993, $\eta^{2}=0.002$, negligible effect size); or differences in the proportion of neurons relative to the overall number of cells (Figure 5c, one-way ANOVA with F(3,44)=1.312, p=0.282, $\eta^{2}=0.082$, moderate effect size), indicating that glial cells were not induced to proliferate. Overall, these findings show that primary neuronal cell cultures over-expressing P301L tau do not have altered cell viability, even when seeded with pTau.

### 3.4. Activity and Connectivity in Neuronal Cultures Treated with P301S Sarkosyl-Insoluble Fraction

We investigated whether mouse primary cortical cultures exhibited significant alterations in both dynamics and effective connectivity when pTau was administered into the culture medium. As described in Methods (protocol I), data were obtained from calcium imaging spontaneous activity recordings at different developmental time points, from DIV 7 to DIV 16 every approximately two days (shown in the Supplementary Material), with detailed comparisons between experimental conditions performed at DIV 12. This time point was selected as optimal for analysis since cultures at earlier stages displayed weak and irregular activity, whereas cultures at later stages—particularly by DIV 16—could exhibit signs of intrinsic degradation that were independent of treatment.

#### 3.4.1. P301S (+/−) Sarkosyl-Insoluble Fraction Does Not Strongly Alter Network Dynamics

**Figure 6:**
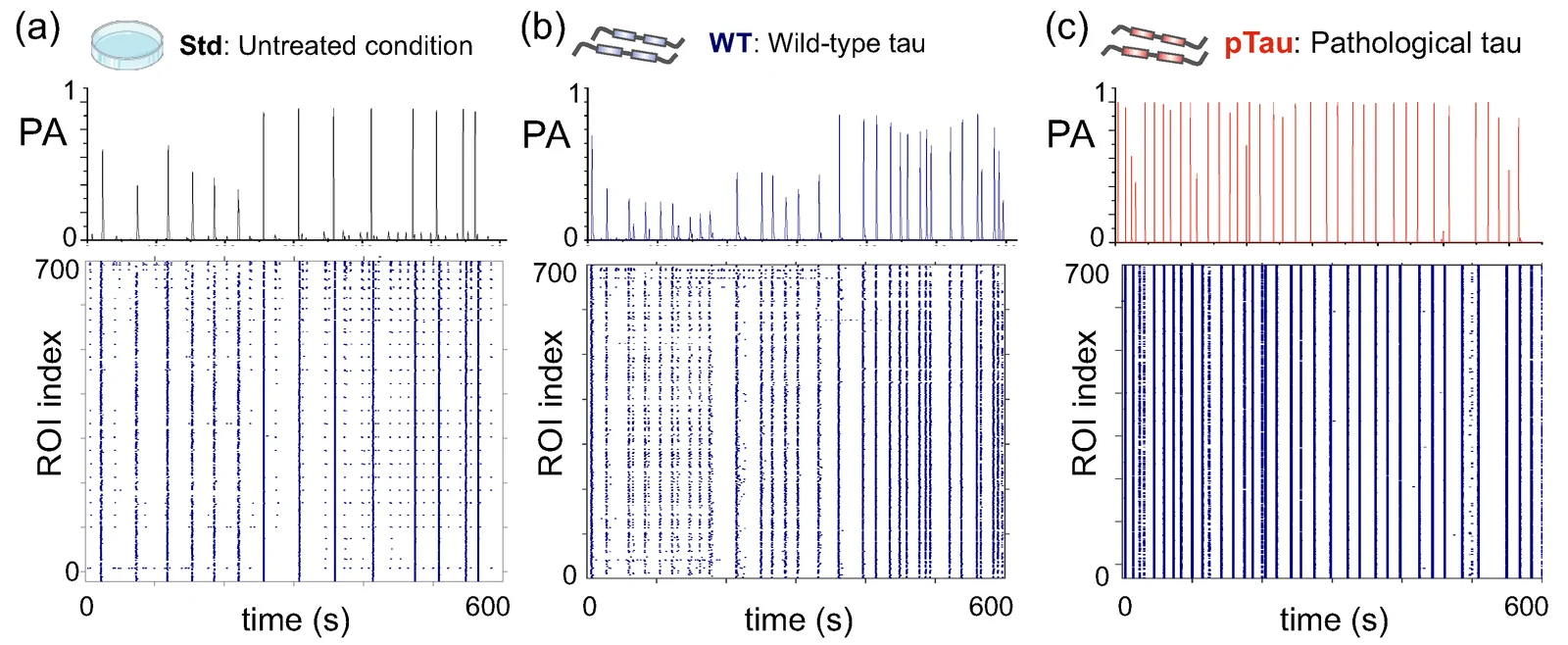
Illustrative spontaneous activity data of neuronal cultures treated with P301S sarkosyl-insoluble fraction at DIV 12. The plots show examples of raster plots (**bottom**) and corresponding population activity PA (**top**) for the three conditions studied at DIV 12: (**a**) Untreated cultures (Std), (**b**) P301S (−/−) sarkosyl-insoluble extracts (WT), and (**c**) P301S (+/−) sarkosyl-insoluble extracts (pTau). High amplitude peaks in PA reflect strong network bursting.

Calcium fluorescence recordings of spontaneous activity in the three conditions considered (Std, WT and pTau) were analyzed and the detected neuronal activity events were plotted in the form of raster plots. Figure 6 shows illustrative raster plots and the corresponding network population activity (PA) for each of the explored conditions at DIV 12, i.e., one week post-treatment. While raster plots depict the temporal structure of activity across individual neurons, the PA quantifies the fraction of neurons that become active within short time windows. Peaks in PA therefore reflect network bursts, a characteristic feature of neuronal cultures grown in flat two-dimensional substrates [32]. At DIV 12, Std and WT cultures (Figure 6a,b) displayed similar firing patterns, with comparable PA variations and a similar number of bursting events. In contrast, pTau cultures (Figure 6c) exhibited markedly higher PA values approaching 1, along with a pronounced increase in burst frequency. This was further reflected in the inter-burst interval (IBI), which was reduced by nearly half in the pTau condition relative to Std and WT cultures. The strengthening of collective activity and the easiness for burst generation suggest that pTau cultures entered a hyperexcitable state in which bursting activity was favored.

**Figure 7:**
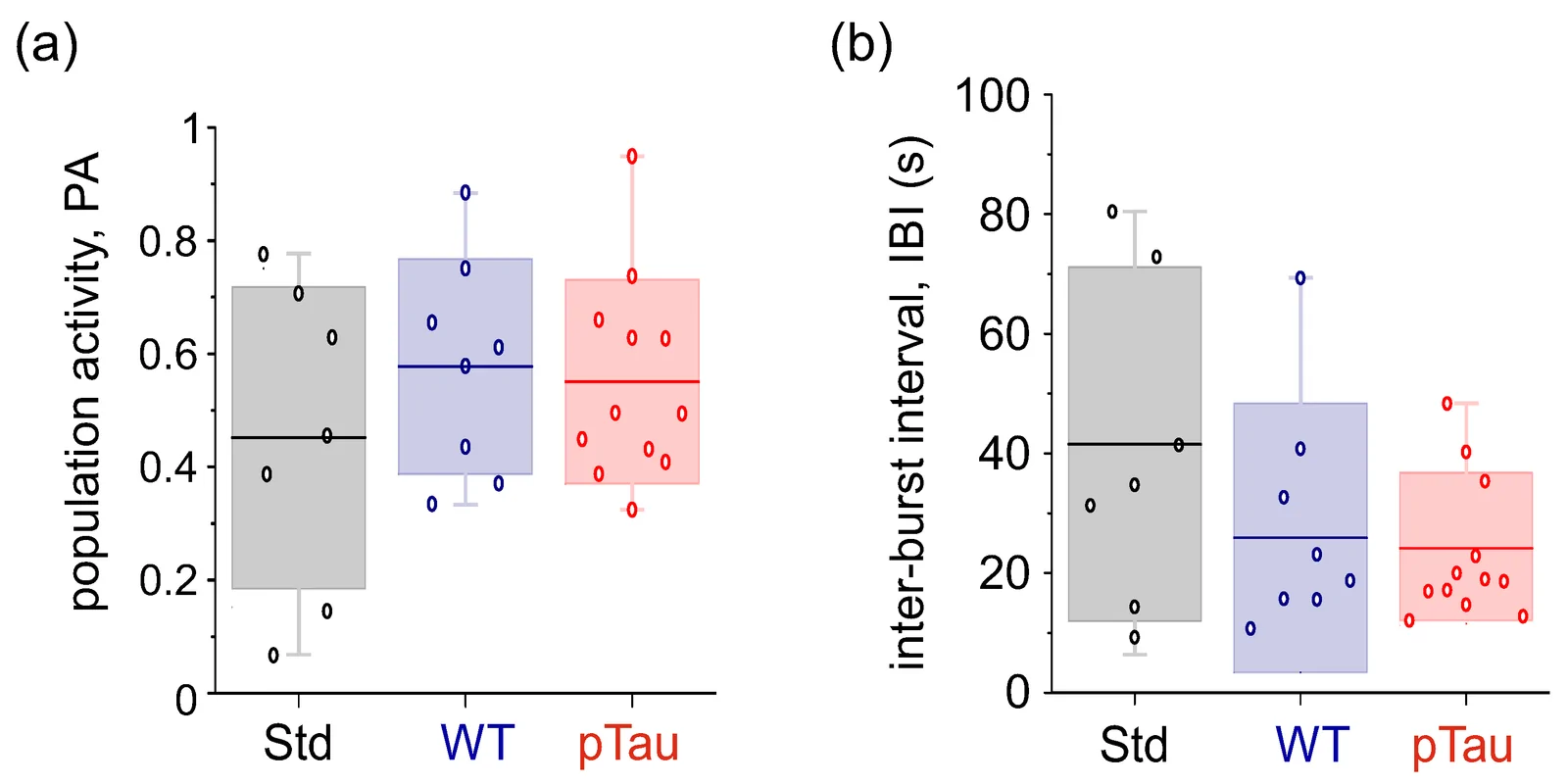
Statistics of dynamic descriptors in cultures treated with P301S sarkosyl-insoluble fraction at DIV 12. (**a**) Box plots showing the distribution of PA values for independent experimental replicates (colored circles) of a given condition at DIV 12. (**b**) Corresponding distribution of IBI values for the different replicates of a given condition at DIV 12. In the box plots, horizontal bars indicate the mean, colored boxes represent mean ± SD, and whiskers the minimum and maximum values. Sample size is n=7 for Std, n=8 for WT and n=12 for pTau condition. No statistically significant differences were detected between conditions (ANOVA followed by Tukey’s post-hoc test).

To assess whether such a possible state of hyperexcitability in pTau cultures—suggested by their elevated PA values—was reproducible, we pooled data from all replicates of each condition at DIV 12, as shown in Figure 7a. The averaged PA evolution from DIV 7 to DIV 16 is presented in Supplementary Figure S2. At DIV 12, although pTau cultures had on average higher PA values than Std, no statistically significant differences were detected across conditions ($p_{\mathrm{Std}\text{-}\mathrm{pTau}}=0.548$, $p_{\mathrm{WT}\text{-}\mathrm{pTau}}=0.984$ and $p_{\mathrm{Std}\text{-}\mathrm{WT}}=0.498$), with a moderate effect size ($\eta^{2}=0.063$). On the other hand, analysis of the average IBI at DIV 12 including different replicates (Figure 7b) revealed a decrease from approximately 40 s in Std cultures to about 25 s in pTau cultures, indicative of an increased burst frequency in the pTau condition. However, statistical analysis did not reveal significant differences between conditions ($p_{\mathrm{Std}\text{-}\mathrm{pTau}}=0.154$, $p_{\mathrm{WT}\text{-}\mathrm{pTau}}=0.835$, $p_{\mathrm{Std}\text{-}\mathrm{WT}}=0.429$), with a strong effect size ($\eta^{2}=0.135$). The averaged IBI evolution from DIV 7 to DIV 16 for all conditions is shown in Supplementary Figure S3.

**Figure 8:**
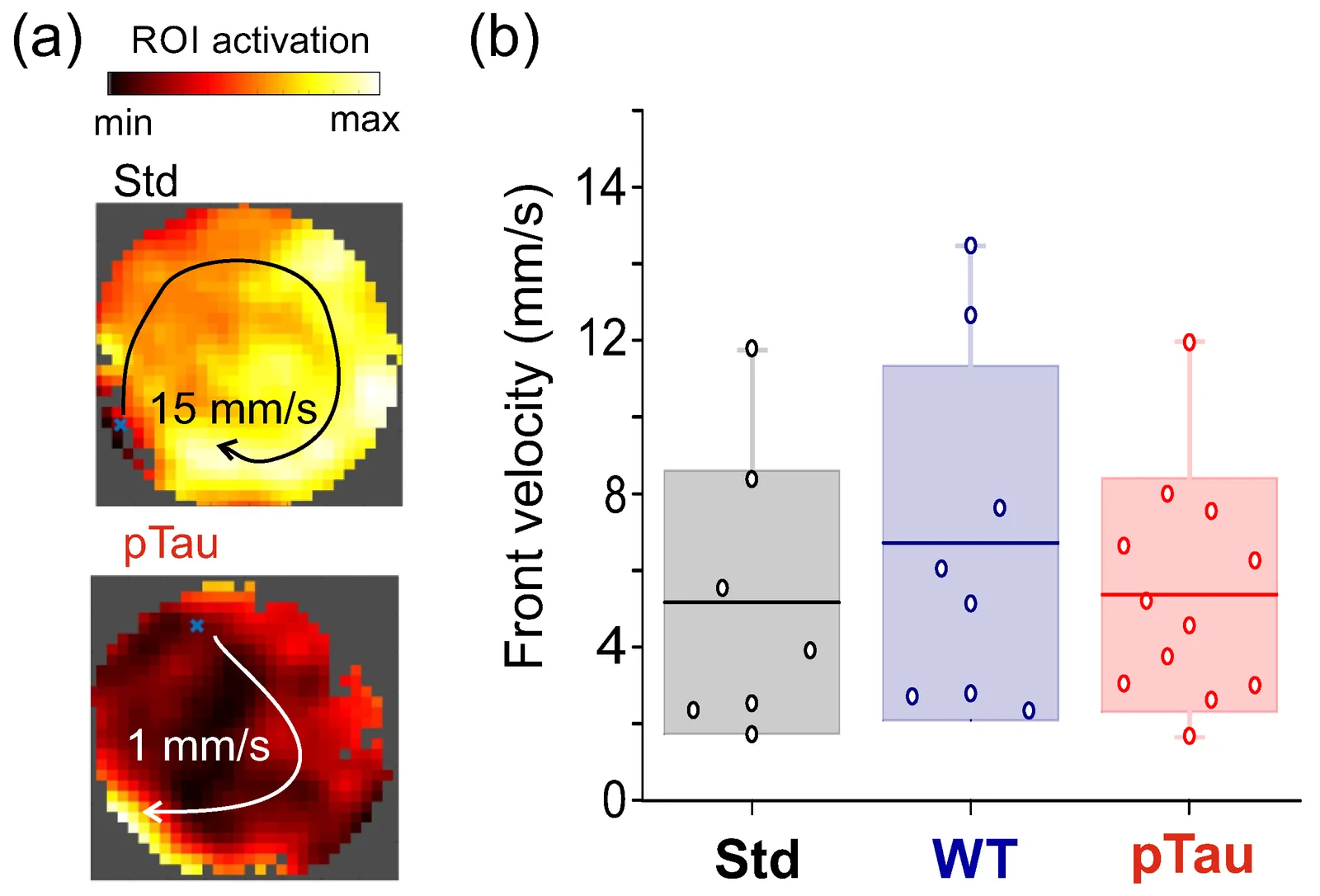
Analysis of activity propagation velocity in cultures treated with the P301S sarkosyl-insoluble fraction at DIV 12. (**a**) Comparison of the evolution of propagating fronts for the Std and pTau conditions. (**b**) Distribution of mean propagation velocity values per culture across the three conditions at DIV 12. Colored circles represent independent experimental replicates, horizontal bars indicate the mean, colored boxes the mean ± SD, and whiskers the minimum and maximum values. Sample size is n=7 for Std, n=8 for WT and n=12 for pTau condition. No statistically significant differences were detected between conditions (ANOVA followed by Tukey’s post-hoc test).

To complete the analysis of dynamical changes, we computed the velocity of network burst propagation in the neuronal cultures. The velocity is a useful descriptor since the higher the connectivity among neurons, the faster the propagation of information across the network [41]. Thus, the velocity provides an indirect dynamical measure of possible network alterations upon pTau treatment. As described in Methods, the velocity was computed by analyzing, for each burst, the activation time of the regions of interest in relation to their spatial location. Figure 8a shows spatiotemporal patterns of burst propagation and the extracted velocities, whereas Figure 8b compares the distribution of velocities across replicates for the three conditions at DIV 12. No significant statistical differences were observed ($p_{\mathrm{Std}\text{-}\mathrm{WT}}=0.553$, $p_{\mathrm{Std}\text{-}\mathrm{pTau}}=0.937$ and $p_{\mathrm{WT}\text{-}\mathrm{pTau}}=0.703$), with a moderate effect size ($\eta^{2}=0.045$). The evolution of replicates-averaged propagation velocity along DIV for the three conditions is presented in Supplementary Figure S4.

Taken together, the results suggest a mild tendency for pTau cultures to exhibit a hyperexcited state compared to their Std counterparts, as indicated by stronger bursting events, i.e., higher PA values. However, when all experimental replicates are considered and multiple descriptors are evaluated, these effects do not reach statistical significance, limiting the strength of any conclusions.

#### 3.4.2. Presence of P301S Extracellular pTau Does Not Alter Effective Connectivity

To provide additional analyses to determine whether pTau altered network formation and functional organization, we carried out a network analysis of the spontaneous activity recordings to extract effective connectivity matrices and key network topological traits.

**Figure 9:**
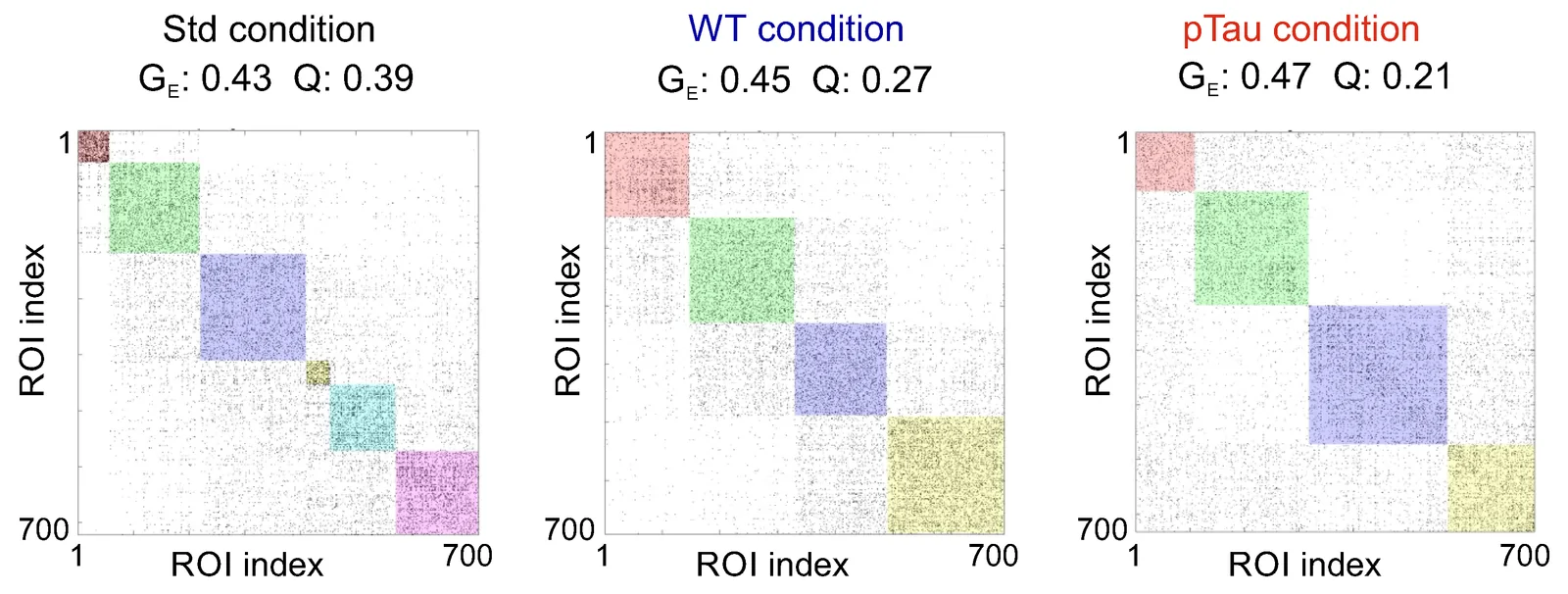
Connectivity in cultures treated with P301S sarkosyl-insoluble fraction at DIV 12. Illustrative connectivity matrices for Std, WT and pTau conditions at DIV 12, obtained from the effective connectivity analysis of a representative raster plot for each condition. In the matrices, black dots indicate effective connections among ROIs, and colored boxes denote functional communities. The network metrics $G_{E}$ and *Q* indicate, respectively, the global efficiency and the community statistic for each condition.

Figure 9 shows examples of effective connectivity matrices for the three conditions. All of them exhibited a similar functional behavior with a comparable number of communities and interconnectivity, with a global efficiency $G_{E}≃0.45$ in the three conditions. Thus, both healthy and pathological cultures established effective connections and overall network communication patterns that appeared largely indistinguishable. The only difference that could be observed among conditions was in the community statistic *Q*, which was higher in the Std condition ($Q_{\mathrm{Std}}≃0.39$) than in the WT ($Q_{\mathrm{WT}}≃0.27$) and pTau ($Q_{\mathrm{pTau}}≃0.21$). Conceptually, this higher presence of communities indicates a greater segregation of network communication in Std cultures, with neuronal interactions occurring more frequently within communities rather than across the network, whereas for WT and pTau, the behavior was characteristic of a more integrated network organization.

**Figure 10:**
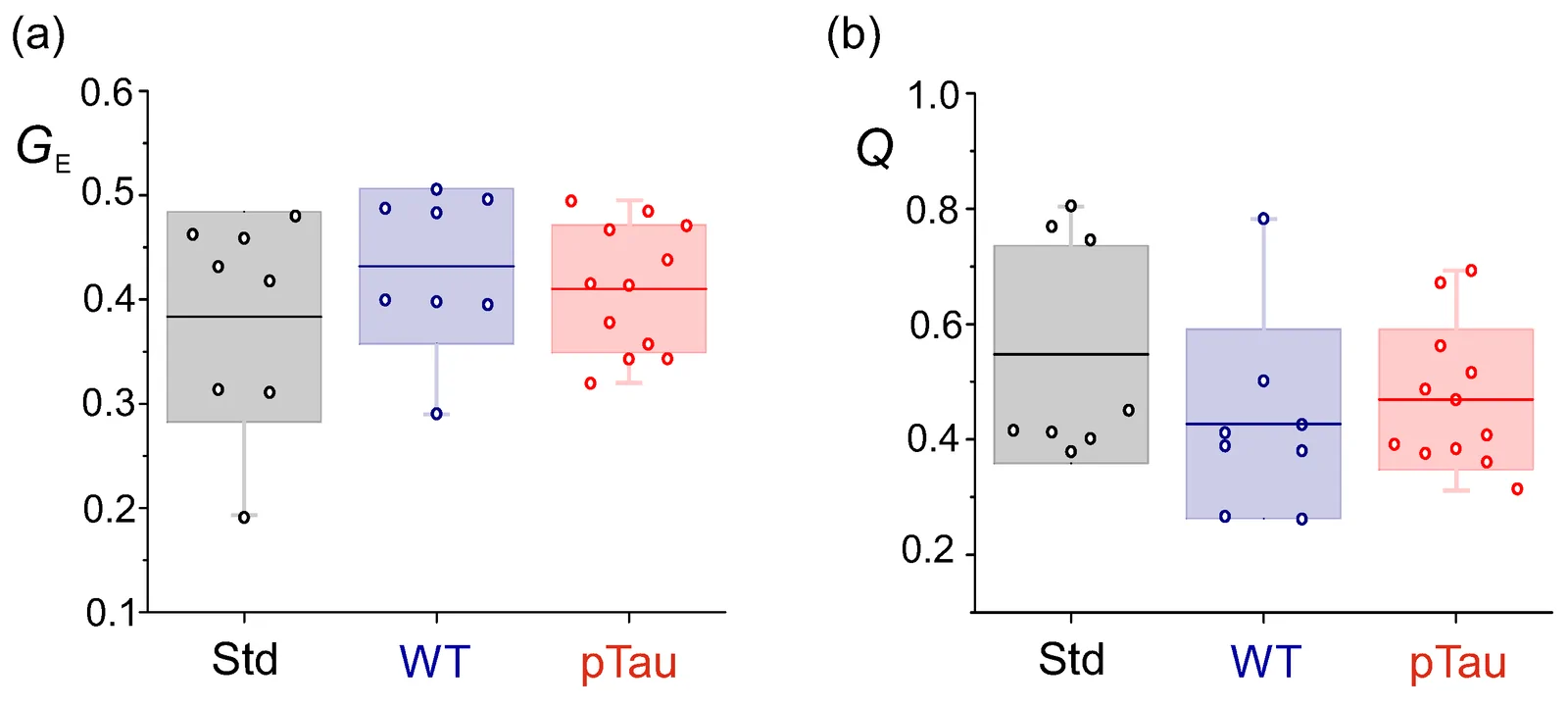
Network metrics in neuronal cultures expressing P301L human tau and subsequently treated with P301S sarkosyl-insoluble fraction at DIV 12. (**a**) Distribution of $G_{E}$ values for each culture condition and replicate at DIV 12. (**b**) Corresponding distribution of *Q* values. Colored circles represent independent experimental replicates, horizontal bars indicate the mean, colored boxes the mean ± SD, and whiskers the minimum and maximum values. Sample size is n=8 for Std, n=8 for WT and n=12 for pTau condition. No statistically significant differences were detected between conditions (ANOVA followed by Tukey’s post-hoc test).

To determine whether the increased network integration observed in pTau cultures—consistent with a state of enhanced communication associated with bursting activity—was reproducible across experiments, we compared the distributions of $G_{E}$ (Figure 10a) and *Q* values (Figure 10b) at DIV 12 for all experimental replicates. Both metrics followed similar trends across conditions, and no statistically significant differences were detected (for $G_{E}$, $p_{\mathrm{Std}\text{-}\mathrm{WT}}=0.483$, $p_{\mathrm{Std}\text{-}\mathrm{pTau}}=0.709$ and $p_{\mathrm{WT}\text{-}\mathrm{pTau}}=0.879$, moderate effect size $\eta^{2}=0.053$; for *Q*, $p_{\mathrm{Std}\text{-}\mathrm{WT}}=0.285$, $p_{\mathrm{Std}\text{-}\mathrm{pTau}}=0.542$ and $p_{\mathrm{WT}\text{-}\mathrm{pTau}}=0.805$, moderate-strong effect size $\eta^{2}=0.090$). Consistent with these findings, the data do not provide evidence that pTau treatment increases network-wide communication. The evolution along DIV for both $G_{E}$ and *Q* is provided in Supplementary Figure S5.

### 3.5. Accentuation of Damage in Culture by Incorporating the P301L Mutation

The above results indicated that no significant alterations were detectable in the dynamics or functional organization of pTau cultures. We therefore speculated that the lack of observable effects could be attributed to the fact that the neurons in our system were young and wild-type, whereas it is well established that aging represents one of the major risk factors for sporadic forms of AD and other neurological disorders [42,43]. In an effort to accelerate damage in in vitro, we induced overexpression of the P301L mutant tau isoform, thereby predisposing the primary neuronal cultures to a more pathological scenario prior to exposing them to extracellular tau (Section 2, protocol II). In these novel culture conditions, neurons were not only exposed to extracellular pTau but were also capable of generating P301L tau isoforms endogenously. Potential dynamical and functional alterations were then quantified through recordings of spontaneous activity and subsequent analyses.

#### 3.5.1. Neuronal Cultures Expressing P301L Human Tau Exhibit a Tendency Toward More Frequent Bursting

**Figure 11:**
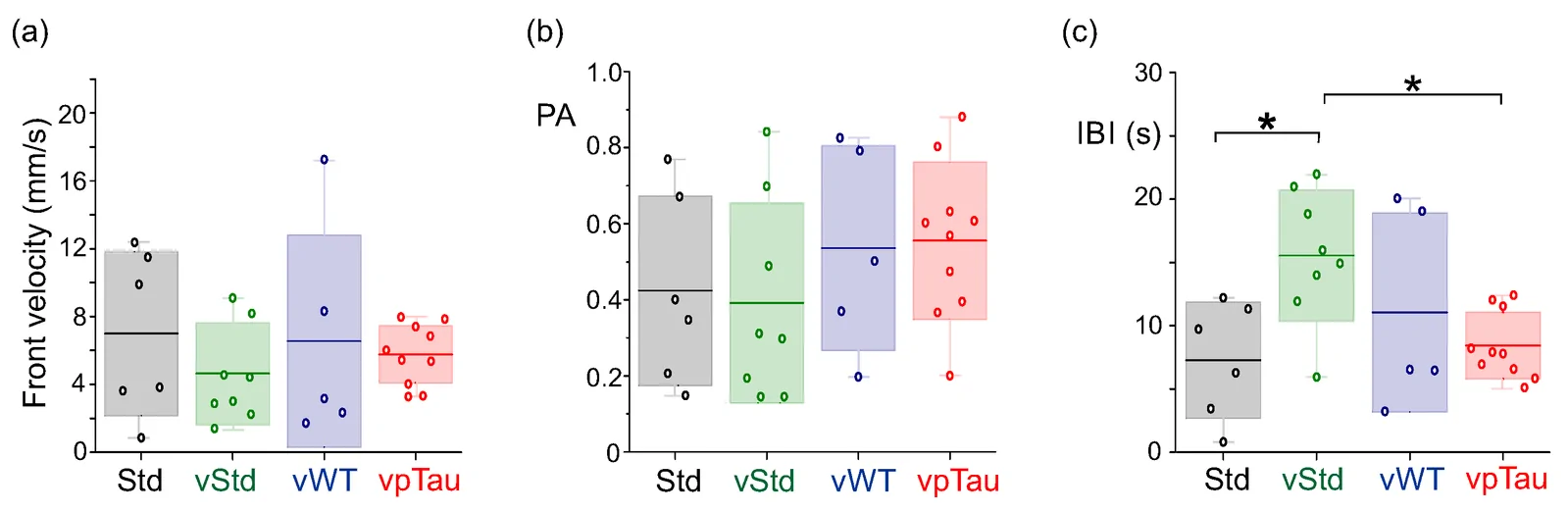
Dynamic observables at DIV 12 in neuronal cultures expressing P301L human tau and subsequently treated with P301S. The panels show box plots comparing statistics across experimental replicates and conditions at DIV 12 for: (**a**) velocity of propagating fronts; (**b**) population activity; (**c**) inter-burst interval. In the box plots, colored circles represent independent experimental replicates, horizontal bars indicate the mean, colored boxes the mean ± SD, and whiskers the minimum and maximum values. Sample size is n=6 for Std, n=8 for vStd, n=5 for vWT and n=10 for vpTau. * p<0.05 (ANOVA followed by Tukey’s post-hoc test).

We explored four new conditions (Std, vStd, vWT and vpTau) and compared their behavior at DIV 12 in terms of velocity of propagating fronts, PA values and IBIs. Supplementary Figure S6 provides illustrative raster plots for each condition, in which only the vpTau exhibited a marked tendency toward more frequent activity. The statistical comparison of conditions across experimental replicates is provided in Figure 11. The evolution of the different culture conditions along development is shown in Supplementary Figure S7.

Velocity analysis at DIV 12 (Figure 11a) revealed no statistically significant differences between conditions ($p_{\mathrm{Std}\text{-}\mathrm{vStd}}=0.642$, $p_{\mathrm{Std}\text{-}\mathrm{vWT}}=0.995$, $p_{\mathrm{Std}\text{-}\mathrm{vpTau}}=0.942$, $p_{\mathrm{vStd}\text{-}\mathrm{vWT}}=0.821$, $p_{\mathrm{vStd}\text{-}\mathrm{vpTau}}=0.883$, and $p_{\mathrm{vWT}\text{-}\mathrm{vpTau}}=0.993$), with a moderate effect size ($\eta^{2}=0.060$). A similar trend was observed for PA (Figure 11b), for which no significant differences were detected among conditions ($p_{\mathrm{Std}\text{-}\mathrm{vStd}}=0.993$, $p_{\mathrm{Std}\text{-}\mathrm{vWT}}=0.866$, $p_{\mathrm{Std}\text{-}\mathrm{vpTau}}=0.735$, $p_{\mathrm{vStd}\text{-}\mathrm{vWT}}=0.706$, $p_{\mathrm{vStd}\text{-}\mathrm{vpTau}}=0.494$, and $p_{\mathrm{vWT}\text{-}\mathrm{vpTau}}=0.999$), with a moderate effect size ($\eta^{2}=0.095$). Finally, analysis of the IBI (Figure 11c) revealed statistically significant differences between Std and vStd ($p_{\mathrm{Std}\text{-}\mathrm{vStd}}=0.0157$) and between vStd and vpTau ($p_{\mathrm{vStd}\text{-}\mathrm{vpTau}}=0.0160$), but not among the remaining conditions ($p_{\mathrm{Std}\text{-}\mathrm{vWT}}=0.499$, $p_{\mathrm{Std}\text{-}\mathrm{vpTau}}=0.974$, $p_{\mathrm{vStd}\text{-}\mathrm{vWT}}=0.405$, and $p_{\mathrm{vWT}\text{-}\mathrm{vpTau}}=0.651$). ANOVA provided a large effect size ($\eta^{2}=0.366$), indicating that experimental condition accounted for approximately 37% of the observed variance. Taken together, vpTau-treated cultures showed a tendency toward reduced IBI values relative to the vStd condition. However, the high variability observed within control conditions limits the interpretation of these effects.

#### 3.5.2. Cultures with Expression of P301L Human Tau Do Not Alter Effective Connectivity

**Figure 12:**
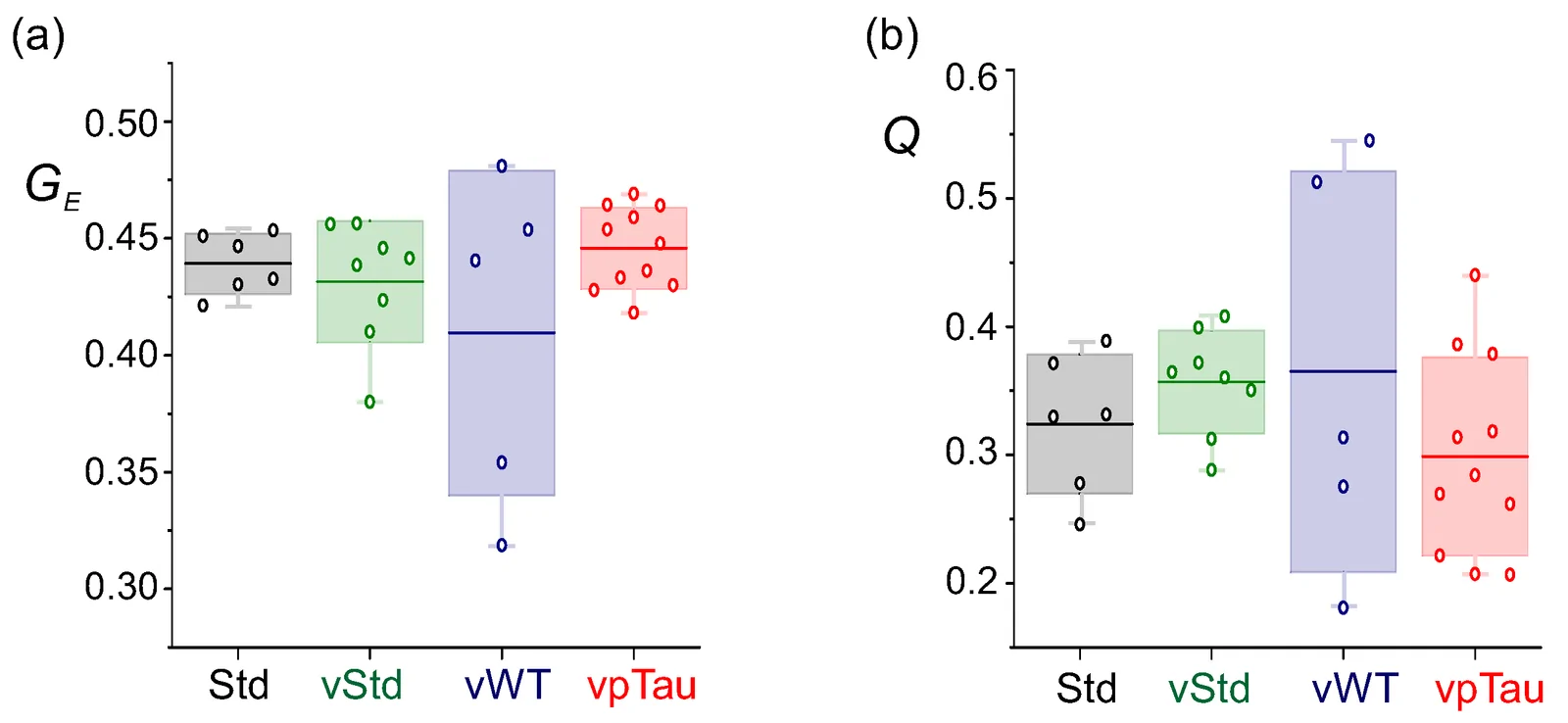
Network metrics at DIV 12 in neuronal cultures expressing P301L human tau and subsequently treated with P301S. The panels show box plots comparing statistics across experimental replicates and conditions at DIV 12 for: (**a**) global efficiency $G_{E}$; (**b**) community statistic *Q*. Colored circles represent independent experimental replicates, horizontal bars indicate the mean, colored boxes the mean ± SD, and whiskers the minimum and maximum values. Sample size is n=6 for Std, n=8 for vStd, n=5 for vWT and n=11 for vpTau. No statistically significant differences were detected between conditions (ANOVA followed by Tukey’s post-hoc test).

The spontaneous activity data for the P301L-expressing cultures were further analyzed to compute the effective connectivity and corresponding $G_{E}$ and *Q* metrics. Comparisons across experimental replicates and conditions at DIV 12 are shown in Figure 12, while the developmental evolution of these metrics is provided in Supplementary Figure S8. At DIV 12, no statistically significant differences between conditions were observed for either $G_{E}$ (Figure 12a) or *Q* (Figure 12b). For $G_{E}$, post-hoc comparisons provided $p_{\mathrm{Std}\text{-}\mathrm{vStd}}=0.955$, $p_{\mathrm{Std}\text{-}\mathrm{vWT}}=0.431$, $p_{\mathrm{Std}\text{-}\mathrm{vpTau}}=0.978$, $p_{\mathrm{vStd}\text{-}\mathrm{vWT}}=0.665$, $p_{\mathrm{vStd}\text{-}\mathrm{vpTau}}=0.735$ and $p_{\mathrm{vWT}\text{-}\mathrm{vpTau}}=0.181$, while the corresponding ANOVA indicated a large effect size ($\eta^{2}=0.152$). For *Q*, post-hoc comparisons provided $p_{\mathrm{Std}\text{-}\mathrm{vStd}}=0.921$, $p_{\mathrm{Std}\text{-}\mathrm{vWT}}=0.842$, $p_{\mathrm{Std}\text{-}\mathrm{vpTau}}=0.931$, $p_{\mathrm{vStd}\text{-}\mathrm{vWT}}=0.992$, $p_{\mathrm{vStd}\text{-}\mathrm{vpTau}}=0.515$ and $p_{\mathrm{vWT}\text{-}\mathrm{vpTau}}=0.456$, with moderate-to-large effect size ($\eta^{2}=0.106$). These results suggest that experimental condition accounted for a non-negligible proportion of the variance in both $G_{E}$ and *Q* despite the absence of statistically significant pairwise differences.

### 3.6. Developmental Trajectories of Dynamic and Connectivity Descriptors

After characterizing network dynamics and connectivity at DIV 12, we examined the longitudinal evolution of these descriptors from DIV 7 to DIV 16 (Supplementary Figures S2–S8) to assess whether pathological tau induced reproducible alterations during network maturation. Although several descriptors exhibited clear developmental trends, condition-dependent effects were generally modest in Protocol I but became progressively apparent in Protocol II, particularly after DIV 12.

For Protocol I, population activity PA was the only descriptor showing a clear condition-dependent effect. Population activity tended to be higher in the Std and WT conditions than in pTau throughout most developmental stages, although these differences were generally modest and varied across DIVs. No significant condition effects were detected at DIV 7, DIV 9, DIV 12, or DIV 14. In contrast, at DIV 16, a one-way ANOVA revealed a significant effect of condition (F(2,18)=4.78, p=0.022, $\eta^{2}=0.347$), with Tukey’s post-hoc test indicating significantly higher PA in WT than in either Std or pTau. Thus, while Std and WT frequently displayed higher PA values than pTau, these differences did not consistently reach statistical significance, with the strongest effect emerging only at late developmental stages.

In contrast, propagation velocity, inter-burst interval, $G_{E}$, and *Q* were primarily characterized by substantial developmental variability and limited discrimination between experimental conditions. Propagation velocity and IBI exhibited large fluctuations across developmental stages, with no stable signature of pTau-induced alterations emerging over time. $G_{E}$ behaved remarkably similarly across conditions, suggesting that overall network integration was largely preserved even with the presence of pathological tau, and with a trend for a gradual increase of $G_{E}$ over time that we ascribe to the strengthening of network integration during maturation. *Q* displayed the clearest developmental trajectory, progressively decreasing from DIV 7 to DIV 14 in all conditions, consistent with the increasing network integration. However, differences among conditions remained modest and changed direction across development.

For Protocol II, PA, IBI, and propagation velocity revealed a progressive divergence of vpTau cultures from the remaining conditions during network maturation. During the early developmental stages (DIV 7 to DIV 9), PA levels were broadly comparable among Std, vStd, vWT, and vpTau cultures. However, from DIV 12 onward, vpTau progressively separated from the other groups, becoming the condition with the highest PA at DIV 16. This increase was paralleled by consistently shorter IBIs, indicative of more frequent burst generation, and by the maintenance of higher propagation velocities at late developmental stages. Although none of these descriptors exhibited statistically significant differences among conditions at any individual DIV, all three metrics (PA, IBIs and velocity) displayed qualitatively similar developmental trajectories. In particular, vpTau cultures tended to show increased PA levels, more frequent bursting, and faster activity propagation relative to the remaining conditions as maturation progressed. Despite the substantial variability observed in all descriptors, the convergence of these trends points toward a progressive shift of vpTau networks toward a more active dynamical regime. This divergence emerged gradually during maturation rather than being present at early developmental stages, suggesting that vpTau modifies the developmental trajectory of network activity rather than inducing an immediate effect.

The connectivity descriptors reinforced this interpretation, although no strong statistical differences among conditions were detected. While $G_{E}$ was broadly comparable among conditions during early development, vpTau cultures displayed progressively higher $G_{E}$ values from DIV 12 onward, reaching the highest levels at DIV 12–16. Conversely, community statistic *Q* consistently decreased in vpTau cultures after DIV 12, yielding the lowest *Q* values among all conditions at later stages. As in protocol I, the concurrent increase in $G_{E}$ and decrease in *Q* suggest that vpTau promotes a transition toward a more integrated network organization. It is worth noting that these connectivity changes emerged during the same developmental period in which the dynamical alterations became apparent, indicating that vpTau reshapes both the activity patterns and the functional organization of neuronal networks during maturation.

## 4. Discussion

The main objective of this work was to develop an in vitro model to assess the impact of extracellular pTau on mouse cortical neuronal cultures using descriptors of network dynamics and functional organization. We used primary neuronal cultures derived from wild-type CD1 mouse embryos, a widely used preparation for investigating the mechanisms underlying sporadic tauopathies. An additional objective was to evaluate whether this simplified in vitro system could provide complementary insight to other experimental approaches, such as animal models that overexpress tau, in which pathology is initiated in vivo and its consequences are typically inferred from cognitive or motor deficits [44,45,46].

### 4.1. Hypothesis for Network Damage and Observations at DIV 12

As a prerequisite for our experiments, we confirmed that sarkosyl-insoluble fractions from P301S (+/−) mouse brains contain pathological tau and insoluble filaments (Figure 4). In AD patients, it has been observed that the progression of pTau aggregate formation correlates with disease progression [47]. In this context, neurons containing insoluble filaments degenerate and liberate reactive forms of tau protein into the extracellular space, known as ghost tangles, which may accumulate and be toxic to healthy neighboring neurons [48,49]. Consistent with this view, extracellular tau has been reported to be toxic and capable of inducing neuronal death in human neuroblastoma cells grown in vitro [50]. Assuming that such toxicity is substantial, the absence of detectable network degradation or cell death in our experiments is therefore unexpected, suggesting that the culture model presented here is not adequate to capture key pathological hallmarks of AD. The absence of detectable signs of network damage was further supported by independent cytotoxicity assays, which showed no reduction in cell viability in cultures exposed to pTau (Figure 5).

Indeed, our hypothesis when designing the in vitro model (using either protocol I or II) was that the known role of pTau in microtubule destabilization and axonal degradation would translate into quantifiable network-wide dynamical changes and connectivity alterations. For this reason, we measured the velocity of propagation of network bursts, since it is tightly dependent on neuronal connectivity and axonal lengths [51,52,53], with higher velocities typically associated with greater average connectivity. We therefore expected that extracellular pTau would substantially reduce the velocity of activity propagation in affected neuronal networks as pTau-induced axonal damage accumulated. However, our data did not support this expectation. At DIV 12, propagation velocities were similar across all experimental conditions. Moreover, analysis of their developmental trajectories (Figures S4 and S7) revealed a gradual decline over time in all groups, although the decrease was less pronounced in pTau-treated cultures. This trend suggests that the observed decrease in velocity is more consistent with typical developmental processes—e.g., pruning of connections after an initial boost in connectivity [54]—rather than with a specific pTau-driven disruption of network circuitry.

We also anticipated that pTau-induced axonal damage would impair population-level activity, as the capacity of a neuronal network to generate bursting events depends critically on connectivity and the rapid exchange of spiking activity among neurons [32,55]. Disruption of these processes was expected to affect spike propagation, activity recruitment, and burst initiation, leading to reduced burst amplitude and increased inter-burst intervals. Contrary to this expectation, no significant drop in PA was observed at DIV 12, and bursting was even facilitated in some pTau-treated conditions, as indicated by a reduced IBI. Similarly, we anticipated alterations in the functional organization of the in vitro networks under pTau treatment through the analysis of $G_{E}$ and *Q*. Despite expectations of reduced efficiency and increased segregation into communities due to impaired connectivity, no significant differences were detected between pTau-treated and control cultures at DIV 12.

### 4.2. Comparison of the Two Protocols and Longitudinal Trends

A comparison of the two protocols highlights an important difference in the effects of pathological tau. In Protocol I, developmental variability largely masked condition-specific effects, with PA representing the only descriptor exhibiting a clear condition-dependent alteration, and only at DIV 16. In contrast, Protocol II displayed a coherent pattern across multiple independent descriptors that emerged after DIV 12. Relative to the other conditions, vpTau cultures tended to exhibit higher PA, shorter IBIs, sustained propagation velocities, increased $G_{E}$, and reduced *Q*. Although these trends were not generally accompanied by strong statistical differences at individual developmental stages, their consistent direction across both dynamical and connectivity descriptors is noteworthy and suggests that vpTau tends to shift neuronal networks toward a more active, integrated, and less modular state during maturation. This trend contrasts with our initial hypothesis of network-wide activity disruption and functional deterioration. Nevertheless, it is consistent with previous in vitro studies, including models of Parkinson’s disease [30], in which pathological conditions were associated with increased network activity and strengthened connectivity.

These findings further indicate that developmental processes exert a strong influence on both network dynamics and topology. However, whereas developmental variability predominated in Protocol I, Protocol II revealed a coordinated alteration of network maturation associated with vpTau. The difficulty in detecting statistically significant differences, despite the convergence of trends across several descriptors, highlights the challenges of monitoring pathology-driven network alterations in neuronal cultures and underscores the importance of considering developmental trajectories when interpreting disease-related phenotypes.

### 4.3. Comparison with Other Models

The concentrations of pTau used in our experiments cannot be directly compared with those present in the human AD brain. First, absolute concentrations of extracellular tau aggregates in the interstitial fluid or cerebrospinal fluid (CSF) of AD patients remain poorly characterized in the literature. Although total tau levels in CSF are typically reported (approximately 200–600 pg/mL in AD versus ≲300 pg/mL in controls), these measurements largely reflect soluble, monomeric tau rather than the sarkosyl-insoluble aggregates used in our study. Second, our preparations consisted of sarkosyl-insoluble material normalized by total protein content (10 μg/mL by BCA assay). Because tau aggregates are structurally heterogeneous and key epitopes are masked within fibrillar assemblies, the true molar concentration of tau monomers within these aggregates cannot be accurately determined without complete denaturation, an approach that would destroy the pathological conformers of interest. Third, the local concentration of tau aggregates at sites of cell-to-cell transfer in vivo is unknown and may differ substantially from bulk CSF or tissue-extract measurements. Therefore, our experimental concentrations were chosen primarily to ensure robust seeding activity within the time frame of the assay, rather than to replicate absolute physiological levels present in AD brain tissue.

We note that there are studies similar to ours in the literature, although with key differences. Stancu et al. [56] treated primary neurons from P301S mice and their wild-type littermates with transgenic truncated human tau fragments, either wild-type or P301L tau. After treatment, changes in calcium fluorescence were quantified using Fura-2 AM. The results showed an increase in both synchronization and oscillation amplitude in neuronal populations derived from P301S mice treated with extracellular tau, whereas no changes were observed in wild-type neurons under the same treatment. In our experiments, wild-type cortical neurons treated with pTau were also unaffected, raising the question of whether wild-type cells are resistant to the presence of extracellular tau. At the time of writing, and to the best of our knowledge, no other study besides that of Stancu et al. has explored the impact of extracellular tau in vitro in wild-type neuronal cultures, with the additional limitation that only measurements at DIV 1 were considered for analysis in Stancu’s work, i.e., only a single time point. It is important to note that Stancu’s study was solely focused on changes in network activity, and no further investigations were conducted, such as analyses of functional organization.

### 4.4. Potential Hyperexcitability Induced by pTau and Its Relation to Human AD Pathology

In light of the absence of overt degradation in our neuronal cultures, together with the longitudinal trends showing a moderate increase in PA values and an accompanying decrease in IBIs, both indicative of enhanced bursting activity, we considered whether pTau-treated cultures may have entered a hyperexcitable state in which neurons were more readily activated. One possibility is that hyperexcitability reflects impaired regulatory processes, such as weakened inhibition or shifts in membrane potential that could facilitate neuronal activity; however, we found no independent evidence to support such interpretations. Previous studies reported that exposure to extracellular tau can elevate intracellular calcium levels [9], potentially altering calcium-fluorescence signals. Yet, our analysis of fluorescence amplitudes across conditions revealed no significant differences, indicating that possible alterations in calcium transport were either absent or not detectable within the constraints of our experimental setup.

Dysregulation of calcium homeostasis has been linked to tau-induced pathology and aberrant excitatory activity, whereas an imbalance between excitation and inhibition is considered a key feature of AD pathology progression [4,57,58]. It has been described that cortical activity regulates the release of endogenous tau under physiological conditions in vivo [15], with elevated excitatory neuronal firing associated with an increase in extracellular tau levels. Yamada et al. [59] linked presynaptic excitatory neuronal activity with the release of tau by neurons in vivo, suggesting that the spread of tauopathy diseases may be an active, synapse-dependent process rather than a passive consequence of neuronal degeneration [60]. In this context, early neuronal hyperactivation observed in AD patients has been proposed as a compensatory response to incipient cognitive decline [61,62].

On the other hand, some authors have questioned the toxic role of pTau and hypothesized a protective role for insoluble tau filaments and neurofibrillary tangles (NFTs). Lee et al. proposed an antioxidant role for the accumulation of phosphorylated tau as a response to oxidative damage [63], which has been reported as one of the main features of AD progression [64,65]. In contrast, Hanger et al. hypothesized that NFT accumulation might result from an effort to restore physiological levels of protein tau available for microtubule binding in neurons when steady-state levels increase [66]. These interpretations, together with observations that extracellular tau often exerts limited toxic effects in wild-type neurons—whether in animal models or in culture—raise important questions about the role of insoluble forms of tau in the development of tauopathies. This aligns with the view, already postulated in the literature, that NFTs may be relatively inert and that the toxicity of pTau relates to the presence of toxic tau intermediates [67,68].

### 4.5. Limitations of Standard In Vitro Models and Future Directions

Conventional in vitro models based on dissociated embryonic neurons cultured on glass substrates impose inherent limitations when modeling neurodegenerative processes. In such systems, cell-secreted neurotoxic factors are diluted into the bulk medium and periodically removed during routine media exchanges, in sharp contrast to the in vivo environment, where highly concentrated extracellular nucleation sites can form. Moreover, pathologies such as tauopathies emerge from complex, multiscale processes involving signaling cascades, synaptic remodeling, and network-level interactions that are extraordinarily difficult to recapitulate in vitro [69].

Primary wild-type neurons, such as those used in our experimental model, may respond to pathological perturbations by engaging compensatory mechanisms that act to preserve a homeostatic “set point” [70]. Consequently, neurons in our cultures may exist in a state of continuous adaptation to extracellular pTau exposure, complicating the detection and monitoring of degradation. Indeed, several in vitro studies using systems comparable to ours have shown that neuronal cultures dynamically adjust their activity to compensate for chemical perturbations [71] or recover from injury by activating adaptive mechanisms that restore network activity toward baseline levels [72,73,74]. One such adaptive mechanism was described by Desai et al., who demonstrated that cultured neurons increase their intrinsic excitability in response to reduced network activity [75]. Synaptic plasticity, which operates through activity-dependent modifications of synaptic weights, is likewise shaped by network-level activity patterns [76]. However, in highly synchronous primary cultures such as those used here, these compensatory changes may be masked—and therefore difficult to quantify—by persistent, network-wide activity, ultimately rendering functional alterations difficult to detect.

We therefore attribute the absence of detectable damage to limitations inherent to our in vitro model, which—beyond the aforementioned constraints—relies on young embryonic neurons. These neurons typically exhibit low baseline synaptic activity and incompletely developed axonal and dendritic arbors, thereby limiting the extent of measurable alterations in structural connectivity and its impact on functional changes. Moreover, immature neurons display a higher degree of plasticity than mature neuronal networks, which may compensate for manifestations of pathology. In addition, tauopathies generally arise and evolve within relatively aged neuronal circuits. Consequently, our monitoring window of approximately two weeks, focused on an early developmental stage, likely limits the ability of the in vitro model to capture disease-relevant network dysfunction. Thus, as an incremental improvement to the current approach, the use of multielectrode arrays could also be considered, as they facilitate long-term neuronal culturing and therefore extended longitudinal monitoring of network activity [77].

In light of these considerations, alternative experimental platforms capable of sustaining neuronal assemblies over extended timescales—such as brain organoids [78,79,80] or human induced pluripotent stem cell (hiPSC)-derived lines [81]—may offer more suitable systems for studying tau-related pathology. For instance, Cesar et al. used cerebral organoids derived from hiPSCs of patients with familial AD to demonstrate that they were able to recapitulate key AD hallmarks such as NFT-like structures [78], providing a more physiologically relevant in vitro model for studying disease mechanisms than existing two-dimensional in vitro systems.

## 5. Conclusions

In this work, we investigated the impact of pathological tau on the dynamics and functional organization of mouse primary neuronal cultures. In a first approach, cultures were exposed to pathological tau isolated from the sarkosyl-insoluble fraction of P301S (+/−) mice, and the evolution of the neuronal networks was monitored during development, with a detailed comparison performed at DIV 12. Neither the endpoint analysis nor the longitudinal characterization of network maturation revealed statistically significant differences in network dynamics or functional traits between conditions. To enhance the potential damaging effects of tau, we implemented a second approach in which cultures were first transduced with human P301L tau and subsequently treated with P301S-derived tau. Under these conditions, we observed a weak increase in bursting activity in cultures treated with pathological tau compared to controls. Longitudinal analyses indicated that this difference emerged gradually during network maturation and persisted over time. Additionally, independent cytotoxicity assays showed no detectable cellular damage, with comparable viability and behavior across all conditions.

We attribute the difficulty in detecting alterations mainly to the in vitro neuronal model employed, which consists of young embryonic neurons developing over approximately sixteen days, a time window that may be insufficient for pathological tau-induced degeneration to emerge at the network level. In addition, the intrinsic bursting activity of two-dimensional neuronal cultures may mask or hinder the detection of more subtle functional changes, while compensatory mechanisms may be activated in such immature networks to counteract perturbations. Overall, our findings highlight the limitations of standard primary neuronal cultures as a model system to study tau-related neurodegenerative processes in vitro. Although longitudinal monitoring allowed us to track network development and detect subtle changes in bursting activity over time, these alterations remained modest and were not accompanied by broader disruptions in network dynamics or functional organization. Our results underscore the need for alternative or complementary experimental platforms that enable longer-term recordings and more sensitive quantification of dynamic and functional alterations, such as brain organoids or hiPSC-derived neuronal culture models.

## Figures and Tables

**Table 1:** Reagents and resources used for the preparation of neuronal cultures, their monitoring, and immunohistochemical characterization.

Reagent or Resource	Source	Catalog Number
Mouse anti-4R tau	Merck Millipore, Burlington, MA, USA	05-804
Mouse anti-β-Tubulin	BioLegend, San Diego, CA, USA	801201
GCaMP6s sensor (AAV9.Syn)	Viral Vector Production Unit (Universitat Autònoma de Barcelona, Spain)	N/A
AAV-P301L	Provided by Dr. José Luis Lanciego (CIMA, Spain)	N/A
Cortical tissue from healthy E16 mouse embryos	Charles River, Wilmington, MA, USA	N/A
NaCl	Thermo Fisher Scientific, Waltham, MA, USA	012314.A3
EGTA	Sigma-Aldrich, Madrid, Spain	324626-25GM
Tris-HCl	Thermo Fisher Scientific, Waltham, MA, USA	15567027
Protease inhibitors	Hoffmann-La Roche, Basel, Switzerland	4693116001
Pierce^TM^ BCA assay kit	Sigma-Aldrich, Madrid, Spain	23225
SDS-PAGE	In house	N/A
Laemmli sample buffer	Bio-Rad, Hercules, CA, USA	1610737
Tween 20 (TTBS)	Sigma-Aldrich, Madrid, Spain	J60497.K3
Azide	Sigma-Aldrich, Madrid, Spain	71289
Horseradish peroxidase	Thermo Fisher Scientific, Waltham, MA, USA	31490
Nitric Acid 65%	AppliChem, Barcelona, Spain	143255.1611
SYLGARD 184 Silicone Elastomer Kit	DWO Europe, Madrid, Spain	01673921
Poly-L-Lysine (PLL)	Sigma-Aldrich, Madrid, Spain	P4707
Glucose	Sigma-Aldrich, Madrid, Spain	G5400-1KG
GlutaMAX	Thermo Fisher Scientific, Waltham, MA, USA	35050038
Gentamicin	Sigma-Aldrich, Madrid, Spain	G1272-10ML
MEM Eagle’s minimum essential medium	Thermo Fisher Scientific, Waltham, MA, USA	21090022
Leibovitz’s L-15 medium	Sigma-Aldrich, Madrid, Spain	L1518-500ML
Horse serum	Thermo Fisher Scientific, Waltham, MA, USA	26050088
Fetal bovine serum	Thermo Fisher Scientific, Waltham, MA, USA	16140071
FUDR	Sigma-Aldrich, Madrid, Spain	F0503
B-27 Plus	Thermo Fisher Scientific, Waltham, MA, USA	15717988

**Table 2:** Additional resources used for the monitoring of neuronal cultures and their analysis.

Resource, Equipment or Software	Source	Catalog Number
4-well plates (1.9 cm^2^ culture area/well)	Thermo Fisher Scientific (Nunc), Waltham, MA, USA	179830
Branson 1800 ultrasonic cleaner	Branson Corporation, Barcelona, Spain	CPX1800H-E
Bunsen burner (Propan)	Campingaz, Lyon, France	CV 300 Plus
Heratherm oven	Thermo Fisher Scientific, Waltham, MA, USA	OMH100
Glass coverslips Ø13 mm	Glaswarenfabrik Karl Hecht, Sondheim vor der Rhön, Denmark	41001113
Inverted fluorescence microscope	Zeiss, Madrid, Spain	Axiovert C25
Camera ORCA Flash 4.0	Hamamatsu, Birkerød, Denmark	C11440-42U40
Syringes for filtering (50 mL)	Fisher Scientific, Waltham, MA, USA	10636531
Syringe filters (0.2 μm, PES)	Thermo Fisher Scientific, Waltham, MA, USA	725-2520
Nalgene Rapid-Flow sterile filter (0.2 μm, 500 mL)	Thermo Fisher Scientific, Waltham, MA, USA	566-0020
Python v3.12	Python Software Foundation, Wilmington, DE, USA	www.python.org (accessed on 2 October 2023)
Hokawo v2.10	Hamamatsu Photonics, Hamamatsu city, Shizuoka, Japan	www.hamamatsu.com (accessed on 8 April 2016)
MATLAB 2018a	MathWorks Inc., Natick, MA, USA	www.mathworks.com (accessed on 15 March 2018)
Netcal v8.6.0	Custom-made software	https://github.com/orlandi (accessed on 10 September 2018)
Gephi v0.10.1	Gephi Consortium (Sciences-Po Médialab), Paris, France	https://gephi.org (accessed on 17 January 2023)
Origin 9.1	OriginLab Corporation, Northampton, MA, USA	www.originlab.com (accessed on 1 September 2014)

## Data Availability

The data presented in this study are openly available in Zenodo at https://zenodo.org/records/18601917 (accessed on 10 February 2026), reference number 10.5281/zenodo.18601917.

## Supplementary Materials

The following supporting information can be downloaded at: https://www.mdpi.com/article/10.3390/biomedicines14081670/s1, Figure S1: P301L human tau transduced neuronal cultures in combination with P301S (+/−) treatment; Figure S2: Population activity along development in cultures treated with P301S sarkosyl-insoluble fraction; Figure S3: Inter-burst interval along development in cultures treated with P301S sarkosyl-insoluble fraction; Figure S4: Front velocity along development in cultures treated with P301S sarkosyl-insoluble fraction; Figure S5: Network metrics along development in cultures treated with P301S sarkosyl-insoluble fraction; Figure S6: Illustrative spontaneous activity data at DIV 12 of neuronal cultures expressing P301L human tau and subsequently treated with P301S sarkosyl-insoluble fraction; Figure S7: Dynamic descriptors along development in neuronal cultures expressing P301L human tau and subsequently treated with P301S sarkosyl-insoluble fraction; Figure S8: Network metrics along development in neuronal cultures expressing P301L human tau and subsequently treated with P301S sarkosyl-insoluble fraction.

## References

[1] Wang Y., Mandelkow E. (2016). Tau in physiology and pathology. Nat. Rev. Neurosci..

[2] Lee V.M., Goedert M., Trojanowski J.Q. (2001). Neurodegenerative tauopathies. Annu. Rev. Neurosci..

[3] Moore K.B., Hung T.J., Fortin J.S. (2023). Hyperphosphorylated tau (p-tau) and drug discovery in the context of Alzheimer’s disease and related tauopathies. Drug Discov. Today.

[4] Maestú F., de Haan W., Busche M.A., DeFelipe J. (2021). Neuronal excitation/inhibition imbalance: Core element of a translational perspective on Alzheimer pathophysiology. Ageing Res. Rev..

[5] Zhang J., Zhang Y., Wang J., Xia Y., Zhang J., Chen L. (2024). Recent advances in Alzheimer’s disease: Mechanisms, clinical trials and new drug development strategies. Signal Transduct. Target. Ther..

[6] Grossman M., Seeley W.W., Boxer A.L., Hillis A.E., Knopman D.S., Ljubenov P.A., Miller B., Piguet O., Rademakers R., Whitwell J.L. (2023). Frontotemporal lobar degeneration. Nat. Rev. Dis. Prim..

[7] Guo T., Noble W., Hanger D.P. (2017). Roles of tau protein in health and disease. Acta Neuropathol..

[8] Götz J., Halliday G., Nisbet R.M. (2019). Molecular pathogenesis of the tauopathies. Annu. Rev. Pathol. Mech. Dis..

[9] Gómez-Ramos A., Díaz-Hernández M., Rubio A., Miras-Portugal M.T., Avila J. (2008). Extracellular tau promotes intracellular calcium increase through M1 and M3 muscarinic receptors in neuronal cells. Mol. Cell. Neurosci..

[10] Bouillet T., Ciba M., Alves C.L., Rodrigues F.A., Thielemann C., Colin M., Buée L., Halliez S. (2022). Revisiting the involvement of tau in complex neural network remodeling: Analysis of the extracellular neuronal activity in organotypic brain slice co-cultures. J. Neural Eng..

[11] Esteras N., Abramov A.Y. (2020). Mitochondrial calcium deregulation in the mechanism of beta-amyloid and tau pathology. Cells.

[12] David D.C., Hauptmann S., Scherping I., Schuessel K., Keil U., Rizzu P., Ravid R., Dröse S., Brandt U., Müller W.E. (2005). Proteomic and functional analyses reveal a mitochondrial dysfunction in P301L tau transgenic mice. J. Biol. Chem..

[13] Dickerson B., Salat D., Greve D., Chua E., Rand-Giovannetti E., Rentz D., Bertram L., Mullin K., Tanzi R., Blacker D. (2005). Increased hippocampal activation in mild cognitive impairment compared to normal aging and AD. Neurology.

[14] Palop J.J., Mucke L. (2010). Synaptic depression and aberrant excitatory network activity in Alzheimer’s disease: Two faces of the same coin?. Neuromol. Med..

[15] Pooler A.M., Phillips E.C., Lau D.H., Noble W., Hanger D.P. (2013). Physiological release of endogenous tau is stimulated by neuronal activity. EMBO Rep..

[16] Sanders D.W., Kaufman S.K., DeVos S.L., Sharma A.M., Mirbaha H., Li A., Barker S.J., Foley A.C., Thorpe J.R., Serpell L.C. (2014). Distinct tau prion strains propagate in cells and mice and define different tauopathies. Neuron.

[17] Clavaguera F., Bolmont T., Crowther R.A., Abramowski D., Frank S., Probst A., Fraser G., Stalder A.K., Beibel M., Staufenbiel M. (2009). Transmission and spreading of tauopathy in transgenic mouse brain. Nat. Cell Biol..

[18] Frost B., Diamond M.I. (2010). Prion-like mechanisms in neurodegenerative diseases. Nat. Rev. Neurosci..

[19] Keller J.M., Frega M. (2019). Past, present, and future of neuronal models in vitro. In Vitro Neuronal Networks: From Culturing Methods to Neuro-Technological Applications.

[20] Soriano J. (2023). Neuronal Cultures: Exploring Biophysics, Complex Systems, and Medicine in a Dish. Biophysica.

[21] Calafate S., Buist A., Miskiewicz K., Vijayan V., Daneels G., De Strooper B., de Wit J., Verstreken P., Moechars D. (2015). Synaptic contacts enhance cell-to-cell tau pathology propagation. Cell Rep..

[22] Wu J.W., Herman M., Liu L., Simoes S., Acker C.M., Figueroa H., Steinberg J.I., Margittai M., Kayed R., Zurzolo C. (2013). Small misfolded Tau species are internalized via bulk endocytosis and anterogradely and retrogradely transported in neurons. J. Biol. Chem..

[23] Slanzi A., Iannoto G., Rossi B., Zenaro E., Constantin G. (2020). In vitro models of neurodegenerative diseases. Front. Cell Dev. Biol..

[24] Allen B., Ingram E., Takao M., Smith M.J., Jakes R., Virdee K., Yoshida H., Holzer M., Craxton M., Emson P.C. (2002). Abundant tau filaments and nonapoptotic neurodegeneration in transgenic mice expressing human P301S tau protein. J. Neurosci..

[25] Soriano J., Rodríguez Martínez M., Tlusty T., Moses E. (2008). Development of input connections in neural cultures. Proc. Natl. Acad. Sci. USA.

[26] Puangmalai N., Bhatt N., Montalbano M., Sengupta U., Gaikwad S., Ventura F., McAllen S., Ellsworth A., Garcia S., Kayed R. (2020). Internalization mechanisms of brain-derived tau oligomers from patients with Alzheimer’s disease, progressive supranuclear palsy and dementia with Lewy bodies. Cell Death Dis..

[27] Orlandi J.G., Fernández-García S., Comella-Bolla A., Masana M., Barriga G.G.D., Yaghoubi M., Kipp A., Canals J.M., Colicos M.A., Davidsen J. (2017). NETCAL: An Interactive Platform for Large-Scale, NETwork and Population Dynamics Analysis of CALcium Imaging Recordings, version 7.0.0.

[28] Grewe B.F., Langer D., Kasper H., Kampa B.M., Helmchen F. (2010). High-speed in vivo calcium imaging reveals neuronal network activity with near-millisecond precision. Nat. Methods.

[29] Montalà-Flaquer M., López-León C.F., Tornero D., Houben A.M., Fardet T., Monceau P., Bottani S., Soriano J. (2022). Rich dynamics and functional organization on topographically designed neuronal networks in vitro. iScience.

[30] Carola G., Malagarriga D., Calatayud C., Pons-Espinal M., Blasco-Agell L., Richaud-Patin Y., Fernandez-Carasa I., Baruffi V., Beltramone S., Molina E. (2021). Parkinson’s disease patient-specific neuronal networks carrying the LRRK2 G2019S mutation unveil early functional alterations that predate neurodegeneration. npj Park. Dis..

[31] Fernández-García S., Orlandi J.G., García-Díaz Barriga G., Rodríguez M., Masana M., Soriano J., Alberch J. (2020). Deficits in coordinated neuronal activity and network topology are striatal hallmarks in Huntington’s disease. BMC Biol..

[32] Orlandi J.G., Soriano J., Alvarez-Lacalle E., Teller S., Casademunt J. (2013). Noise focusing and the emergence of coherent activity in neuronal cultures. Nat. Phys..

[33] Stetter O., Battaglia D., Soriano J., Geisel T. (2012). Model-free reconstruction of excitatory neuronal connectivity from calcium imaging signals. PLoS Comput. Biol..

[34] Ludl A.A., Soriano J. (2020). Impact of physical obstacles on the structural and effective connectivity of in silico neuronal circuits. Front. Comput. Neurosci..

[35] Tibau E., Ludl A.A., Ruediger S., Orlandi J.G., Soriano J. (2018). Neuronal spatial arrangement shapes effective connectivity traits of in vitro cortical networks. IEEE Trans. Netw. Sci. Eng..

[36] Rubinov M., Sporns O. (2010). Complex network measures of brain connectivity: Uses and interpretations. Neuroimage.

[37] Latora V., Marchiori M. (2003). Economic small-world behavior in weighted networks. Eur. Phys. J. B-Condens. Matter Complex Syst..

[38] Blondel V.D., Guillaume J.L., Lambiotte R., Lefebvre E. (2008). Fast unfolding of communities in large networks. J. Stat. Mech. Theory Exp..

[39] Holmes B.B., Furman J.L., Mahan T.E., Yamasaki T.R., Mirbaha H., Eades W.C., Belaygorod L., Cairns N.J., Holtzman D.M., Diamond M.I. (2014). Proteopathic tau seeding predicts tauopathy in vivo. Proc. Natl. Acad. Sci. USA.

[40] Kamath T.V., Klickstein N., Commins C., Fernandes A.R., Oakley D.H., Frosch M.P., Hyman B.T., Dujardin S. (2021). Kinetics of tau aggregation reveals patient-specific tau characteristics among Alzheimer’s cases. Brain Commun..

[41] Jacobi S., Soriano J., Moses E. (2010). BDNF and NT-3 increase velocity of activity front propagation in unidimensional hippocampal cultures. J. Neurophysiol..

[42] Braak H., Braak E. (1991). Neuropathological stageing of Alzheimer-related changes. Acta Neuropathol..

[43] Frost B., Götz J., Feany M.B. (2015). Connecting the dots between tau dysfunction and neurodegeneration. Trends Cell Biol..

[44] Xu Q.Q., Yang W., Zhong M., Lin Z.X., Gray N.E., Xian Y.F. (2023). Animal models of Alzheimer’s disease: Preclinical insights and challenges. Acta Mater. Medica.

[45] Nakai T., Yamada K., Mizoguchi H. (2021). Alzheimer’s disease animal models: Elucidation of biomarkers and therapeutic approaches for cognitive impairment. Int. J. Mol. Sci..

[46] Maruyama M., Shimada H., Suhara T., Shinotoh H., Ji B., Maeda J., Zhang M.R., Trojanowski J.Q., Lee V.M.Y., Ono M. (2013). Imaging of tau pathology in a tauopathy mouse model and in Alzheimer patients compared to normal controls. Neuron.

[47] Delacourte A., David J.P., Sergeant N., Buee L., Wattez A., Vermersch P., Ghozali F., Fallet-Bianco C., Pasquier F., Lebert F. (1999). The biochemical pathway of neurofibrillary degeneration in aging and Alzheimer’s disease. Neurology.

[48] Goedert M. (1999). Filamentous nerve cell inclusions in neurodegenerative diseases: Tauopathies and alpha-synucleinopathies. Philos. Trans. R. Soc. Lond. Ser. B Biol. Sci..

[49] Frost B., Jacks R.L., Diamond M.I. (2009). Propagation of tau misfolding from the outside to the inside of a cell. J. Biol. Chem..

[50] Gómez-Ramos A., Díaz-Hernández M., Cuadros R., Hernández F., Avila J. (2006). Extracellular tau is toxic to neuronal cells. FEBS Lett..

[51] Golomb D., Ermentrout G.B. (1999). Continuous and lurching traveling pulses in neuronal networks with delay and spatially decaying connectivity. Proc. Natl. Acad. Sci. USA.

[52] Bressloff P.C. (2000). Traveling waves and pulses in a one-dimensional network of excitable integrate-and-fire neurons. J. Math. Biol..

[53] Feinerman O., Segal M., Moses E. (2005). Signal propagation along unidimensional neuronal networks. J. Neurophysiol..

[54] Cullen D.K., Gilroy M.E., Irons H.R., LaPlaca M.C. (2010). Synapse-to-neuron ratio is inversely related to neuronal density in mature neuronal cultures. Brain Res..

[55] Hernández-Navarro L., Faci-Lázaro S., Orlandi J.G., Feudel U., Gómez-Gardeñes J., Soriano J. (2021). Noise-driven amplification mechanisms governing the emergence of coherent extreme events in excitable systems. Phys. Rev. Res..

[56] Stancu I.C., Vasconcelos B., Ris L., Wang P., Villers A., Peeraer E., Buist A., Terwel D., Baatsen P., Oyelami T. (2015). Templated misfolding of Tau by prion-like seeding along neuronal connections impairs neuronal network function and associated behavioral outcomes in Tau transgenic mice. Acta Neuropathol..

[57] Palop J.J., Chin J., Roberson E.D., Wang J., Thwin M.T., Bien-Ly N., Yoo J., Ho K.O., Yu G.Q., Kreitzer A. (2007). Aberrant excitatory neuronal activity and compensatory remodeling of inhibitory hippocampal circuits in mouse models of Alzheimer’s disease. Neuron.

[58] Styr B., Slutsky I. (2018). Imbalance between firing homeostasis and synaptic plasticity drives early-phase Alzheimer’s disease. Nat. Neurosci..

[59] Yamada K., Holth J.K., Liao F., Stewart F.R., Mahan T.E., Jiang H., Cirrito J.R., Patel T.K., Hochgräfe K., Mandelkow E.M. (2014). Neuronal activity regulates extracellular tau in vivo. J. Exp. Med..

[60] Kobayashi S., Tanaka T., Soeda Y., Takashima A. (2019). Enhanced tau protein translation by hyper-excitation. Front. Aging Neurosci..

[61] Dickerson B.C., Salat D.H., Bates J.F., Atiya M., Killiany R.J., Greve D.N., Dale A.M., Stern C.E., Blacker D., Albert M.S. (2004). Medial temporal lobe function and structure in mild cognitive impairment. Ann. Neurol..

[62] Targa Dias Anastacio H., Matosin N., Ooi L. (2022). Neuronal hyperexcitability in Alzheimer’s disease: What are the drivers behind this aberrant phenotype?. Transl. Psychiatry.

[63] Lee H.g., Perry G., Moreira P.I., Garrett M.R., Liu Q., Zhu X., Takeda A., Nunomura A., Smith M.A. (2005). Tau phosphorylation in Alzheimer’s disease: Pathogen or protector?. Trends Mol. Med..

[64] Alavi Naini S.M., Soussi-Yanicostas N. (2015). Tau hyperphosphorylation and oxidative stress, a critical vicious circle in neurodegenerative tauopathies?. Oxidative Med. Cell. Longev..

[65] Cassidy L., Fernandez F., Johnson J.B., Naiker M., Owoola A.G., Broszczak D.A. (2020). Oxidative stress in alzheimer’s disease: A review on emergent natural polyphenolic therapeutics. Complement. Ther. Med..

[66] Hanger D.P., Anderton B.H., Noble W. (2009). Tau phosphorylation: The therapeutic challenge for neurodegenerative disease. Trends Mol. Med..

[67] Castellani R.J., Nunomura A., Lee H.g., Perry G., Smith M.A. (2008). Phosphorylated tau: Toxic, protective, or none of the above. J. Alzheimer’s Dis..

[68] Cárdenas-Aguayo M.d.C., Gómez-Virgilio L., DeRosa S., Meraz-Ríos M.A. (2014). The role of tau oligomers in the onset of Alzheimer’s disease neuropathology. ACS Chem. Neurosci..

[69] Palop J.J., Chin J., Mucke L. (2006). A network dysfunction perspective on neurodegenerative diseases. Nature.

[70] Turrigiano G. (2012). Homeostatic synaptic plasticity: Local and global mechanisms for stabilizing neuronal function. Cold Spring Harb. Perspect. Biol..

[71] Estévez-Priego E., Teller S., Granell C., Arenas A., Soriano J. (2020). Functional strengthening through synaptic scaling upon connectivity disruption in neuronal cultures. Netw. Neurosci..

[72] Teller S., Estévez-Priego E., Granell C., Tornero D., Andilla J., Olarte O.E., Loza-Alvarez P., Arenas A., Soriano J. (2020). Spontaneous functional recovery after focal damage in neuronal cultures. Eneuro.

[73] Ayasreh S., Jurado I., López-León C.F., Montalà-Flaquer M., Soriano J. (2022). Dynamic and Functional Alterations of Neuronal Networks In Vitro upon Physical Damage: A Proof of Concept. Micromachines.

[74] Sumi T., Houben A.M., Yamamoto H., Kato H., Katori Y., Soriano J., Hirano-Iwata A. (2025). Modular architecture confers robustness to damage and facilitates recovery in spiking neural networks modeling in vitro neurons. Front. Neurosci..

[75] Desai N.S., Rutherford L.C., Turrigiano G.G. (1999). Plasticity in the intrinsic excitability of cortical pyramidal neurons. Nat. Neurosci..

[76] Fauth M., Wörgötter F., Tetzlaff C. (2015). The formation of multi-synaptic connections by the interaction of synaptic and structural plasticity and their functional consequences. PLoS Comput. Biol..

[77] Jahnke H.G., Krinke D., Seidel D., Lilienthal K., Schmidt S., Azendorf R., Fischer M., Mack T., Striggow F., Althaus H. (2017). A novel 384-multiwell microelectrode array for the impedimetric monitoring of Tau protein induced neurodegenerative processes. Biosens. Bioelectron..

[78] Gonzalez C., Armijo E., Bravo-Alegria J., Becerra-Calixto A., Mays C.E., Soto C. (2018). Modeling amyloid beta and tau pathology in human cerebral organoids. Mol. Psychiatry.

[79] Shimada H., Sato Y., Sasaki T., Shimozawa A., Imaizumi K., Shindo T., Miyao S., Kiyama K., Kondo T., Shibata S. (2022). A next-generation iPSC-derived forebrain organoid model of tauopathy with tau fibrils by AAV-mediated gene transfer. Cell Rep. Methods.

[80] Venkataraman L., Fair S.R., McElroy C.A., Hester M.E., Fu H. (2022). Modeling neurodegenerative diseases with cerebral organoids and other three-dimensional culture systems: Focus on Alzheimer’s disease. Stem Cell Rev. Rep..

[81] Bravo C.P., Giani A.M., Madero-Perez J., Zhao Z., Wan Y., Samelson A.J., Wong M.Y., Evangelisti A., Cordes E., Fan L. (2024). Human iPSC 4R tauopathy model uncovers modifiers of tau propagation. Cell.

