# Supplementary material for: Limited Detectability of Network Functional Alterations in a Tauopathy Model Using Mouse Primary Cortical Cultures
Source: Biomedicines. 2026 Jul 24;14(8):1670. doi: 10.3390/biomedicines14081670 (PMC13510110; doi:10.3390/biomedicines14081670)
Supplement: Supplementary file 1 [file biomedicines-14-01670-s001.zip › biomedicines-4175798-supplementary.pdf]

Supplementary Materials: Limited Detectability of Network Functional Alterations in a Tauopathy Model Using Mouse Primary Cortical Cultures

1. Overview

Here we provide additional immunostaining data and the figures for the evolution of different metrics (derived from neuronal dynamics or effective connectivity) along development, from DIV 7 to DIV 16, for both *protocol I* (experiments with P301S sarkosyl-insoluble fraction) and *protocol II* (experiments with additional AAV-P301L transduction to accentuate damage). Representative raster plots for protocol II are also provided.

2. Supplementary figures

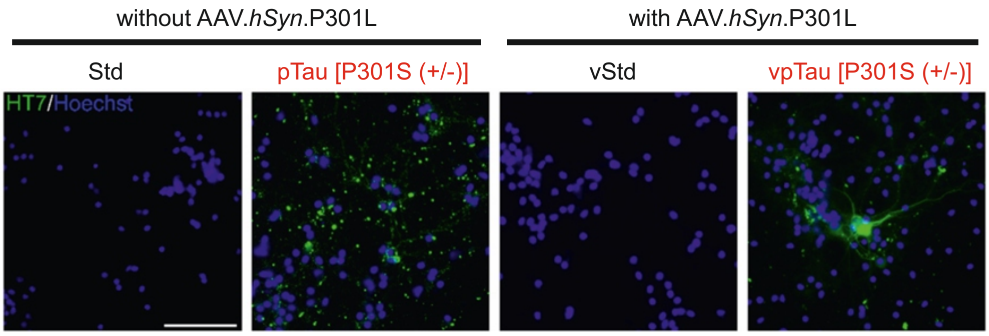

Figure S1. P301L human tau transduced neuronal cultures in combination with P301S (+/-) treatment. [Related to Figure 4 of the main manuscript]. Representative immunostaining images of neuronal cultures with a large field of view, comparing untransduced and AAV.hSyn.P301L transduced neuronal networks at DIV 16, and cultured either in standard conditions (Std and vStd) or subsequently treated with P301S (+/-) (pTau and vpTau). Scale bar is 150 μm.

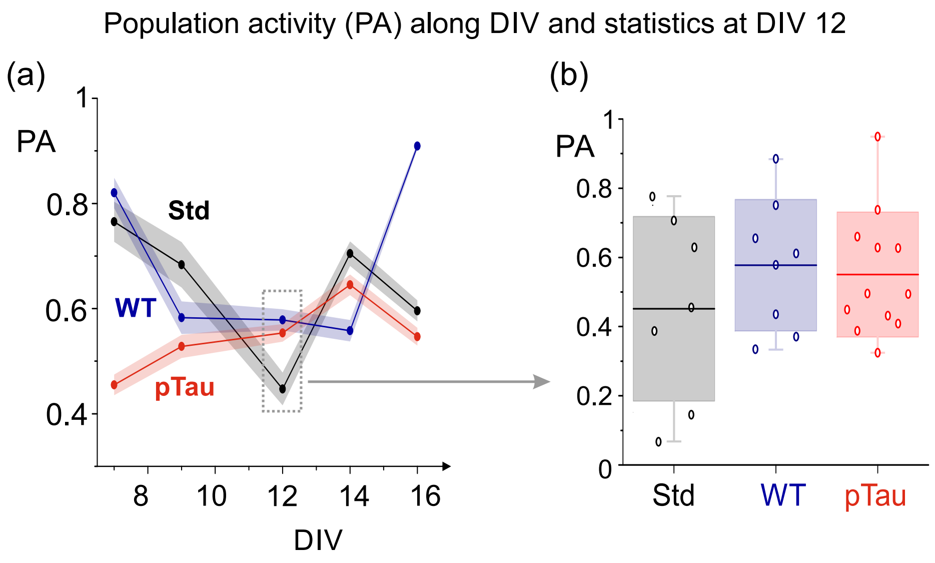

Figure S2. Population activity along development in cultures treated with P301S sarkosyl-insoluble fraction. [Related to Figure 7a of the main manuscript]. **(a)**: Evolution of the population activity (PA) along DIV for the three conditions of 'protocol I' (Std, WT and pTau). Each dot is the average value among experimental replicates, and shadings are standard error. **(b)** Box plots comparing PA statistics across experimental replicates and conditions at DIV 12. Colored circles represent independent experimental replicates, horizontal bars indicate the mean, colored boxes the mean ± SD, and whiskers the minimum and maximum values. Sample size is $n = 7$ for Std, $n = 8$ for WT and $n = 12$ for pTau condition. No statistically significant differences were detected between conditions (ANOVA followed by Tukey's post-hoc test).

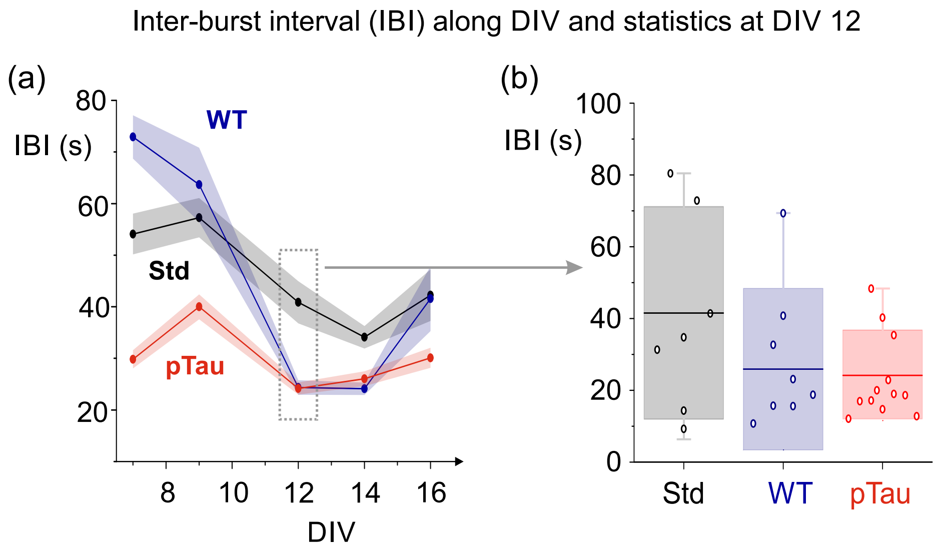

Figure S3. Inter-burst interval along development in cultures treated with P301S sarkosyl-insoluble fraction. [Related to Figure 7a of the main manuscript]. **(a)** Evolution of the inter-burst interval (IBI) along DIV for the three conditions of 'protocol I' (Std, WT and pTau). Each dot is the average value among experimental replicates, and shadings are standard error. **(b)** Box plots comparing IBI statistics across experimental replicates and conditions at DIV 12. Colored circles represent independent experimental replicates, horizontal bars indicate the mean, colored boxes the mean ± SD, and whiskers the minimum and maximum values. Sample size is $n = 7$ for Std, $n = 8$ for WT and $n = 12$ for pTau condition. No statistically significant differences were detected between conditions (ANOVA followed by Tukey's post-hoc test).

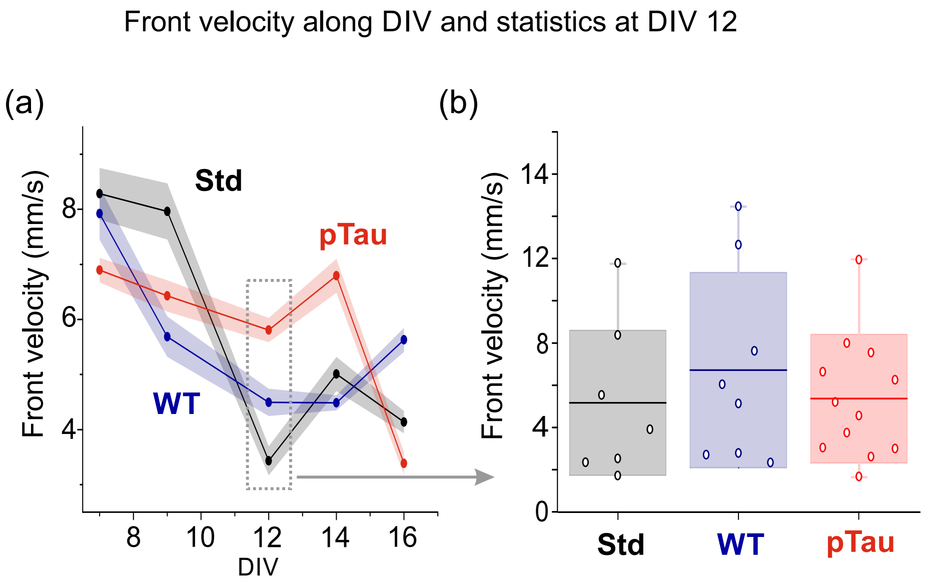

Figure S4. Front velocity along development in cultures treated with P301S sarkosyl-insoluble fraction. [Related to Figure 8 of the main manuscript]. **(a)** Evolution of the front propagation velocity along DIV for the three conditions of 'protocol I' (Std, WT and pTau). Each dot is the average value among experimental replicates, and shadings are standard error. **(b)** Box plots comparing front propagation statistics across experimental replicates and conditions at DIV 12. Colored circles represent independent experimental replicates, horizontal bars indicate the mean, colored boxes the mean ± SD, and whiskers the minimum and maximum values. Sample size is $n = 7$ for Std, $n = 8$ for WT and $n = 12$ for pTau condition. No statistically significant differences were detected between conditions (ANOVA followed by Tukey's post-hoc test).

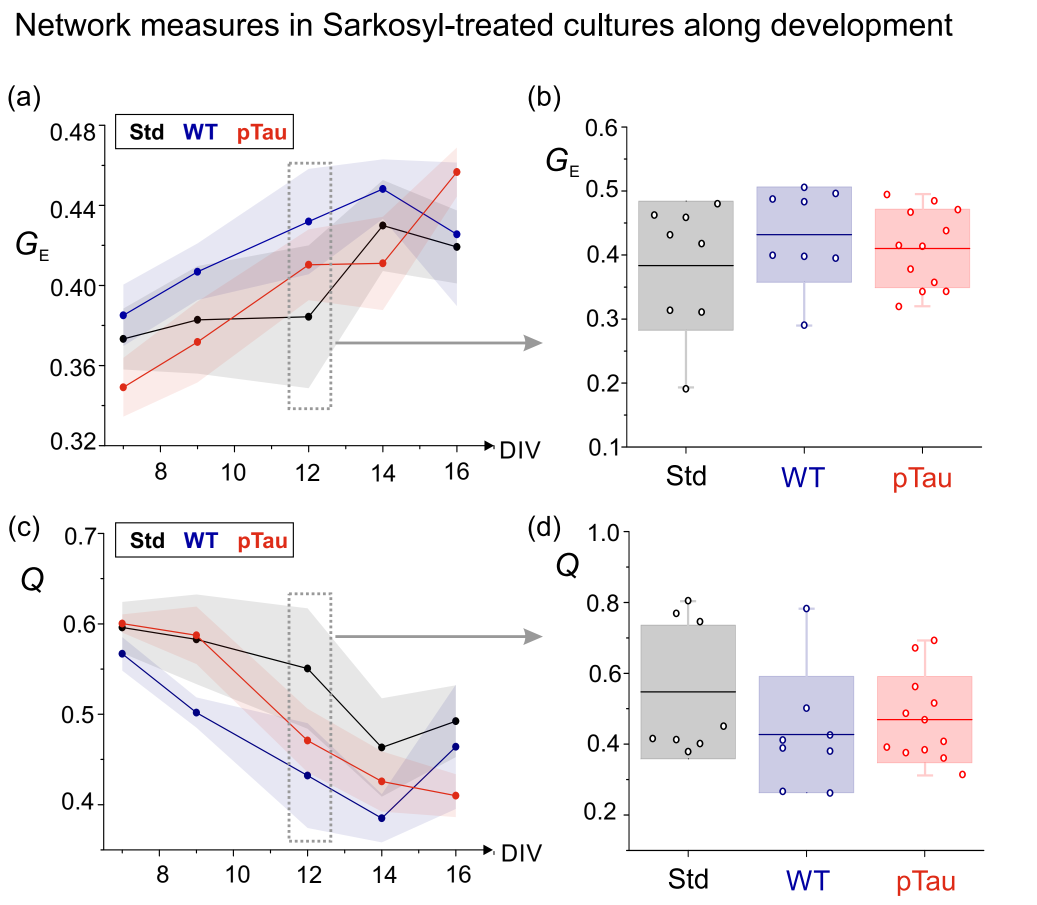

Figure S5. Network metrics along development in cultures treated with P301S sarkosyl-insoluble fraction. [Related to Figure 10 of the main manuscript]. **(a) and (c)** Evolution of average global efficiency G_E and community statistic Q, respectively, along DIV for the three conditions of 'protocol I' (Std, WT and pTau), together with their standard error (color shadings). **(b) and (d)** Corresponding box plots comparing G_E and Q statistics across experimental replicates and conditions at DIV 12. Colored circles represent independent experimental replicates, horizontal bars indicate the mean, colored boxes the mean ± SD, and whiskers the minimum and maximum values. Sample size is $n = 8$ for Std, $n = 8$ for WT and $n = 12$ for pTau condition. No statistically significant differences were detected between conditions (ANOVA followed by Tukey's post-hoc test).

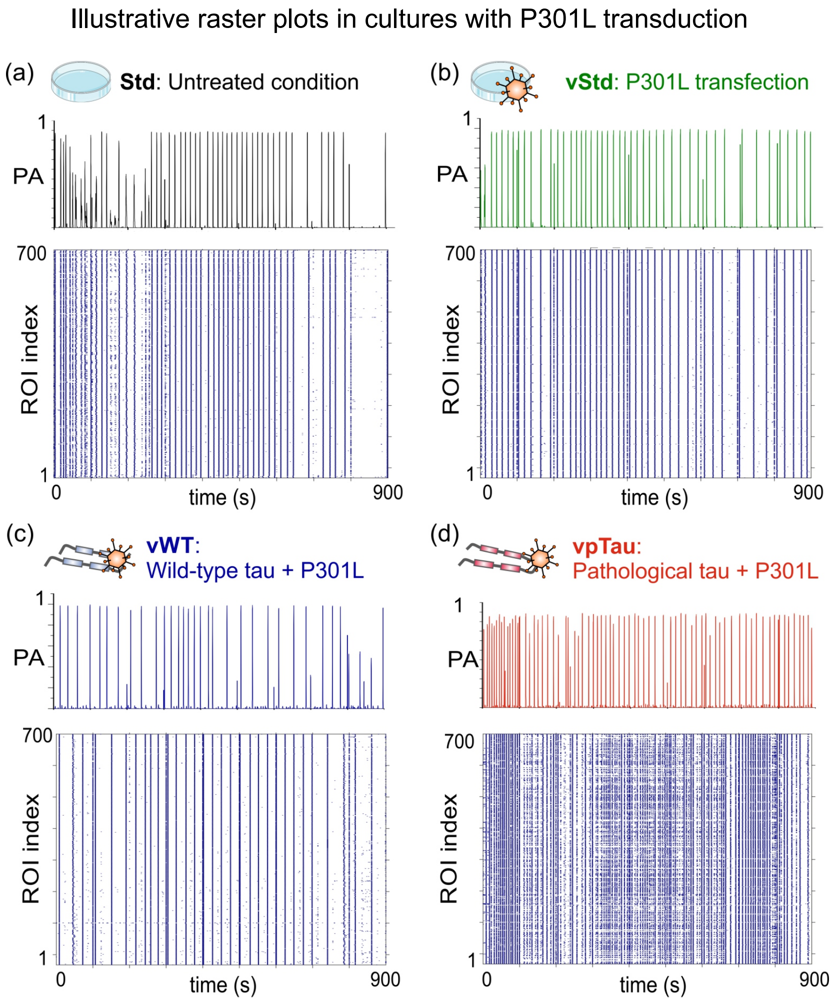

Figure S6. Illustrative spontaneous activity data at DIV 12 of neuronal cultures expressing P301L human tau and subsequently treated with P301S sarkosyl-insoluble fraction. [Related to Figure 11 of the main manuscript]. The panels show raster plots and population activity (PA) quantification for: **(a)** a neuronal culture at DIV 12 without P301L transduction and maintained in standard conditions (Std); **(b)** a culture transduced with P301L and maintained in standard conditions (vStd); **(c)** a culture transduced with P301L and subsequently treated P301S (-/-) wild-type tau (vWT); **(d)** a culture transduced with P301L and subsequently treated P301S (+/-) pathological tau (vpTau). For the latter panel, the presence of pTau increased neuronal bursting, as observed by the higher density of collective activity events in both the raster plot and PA.

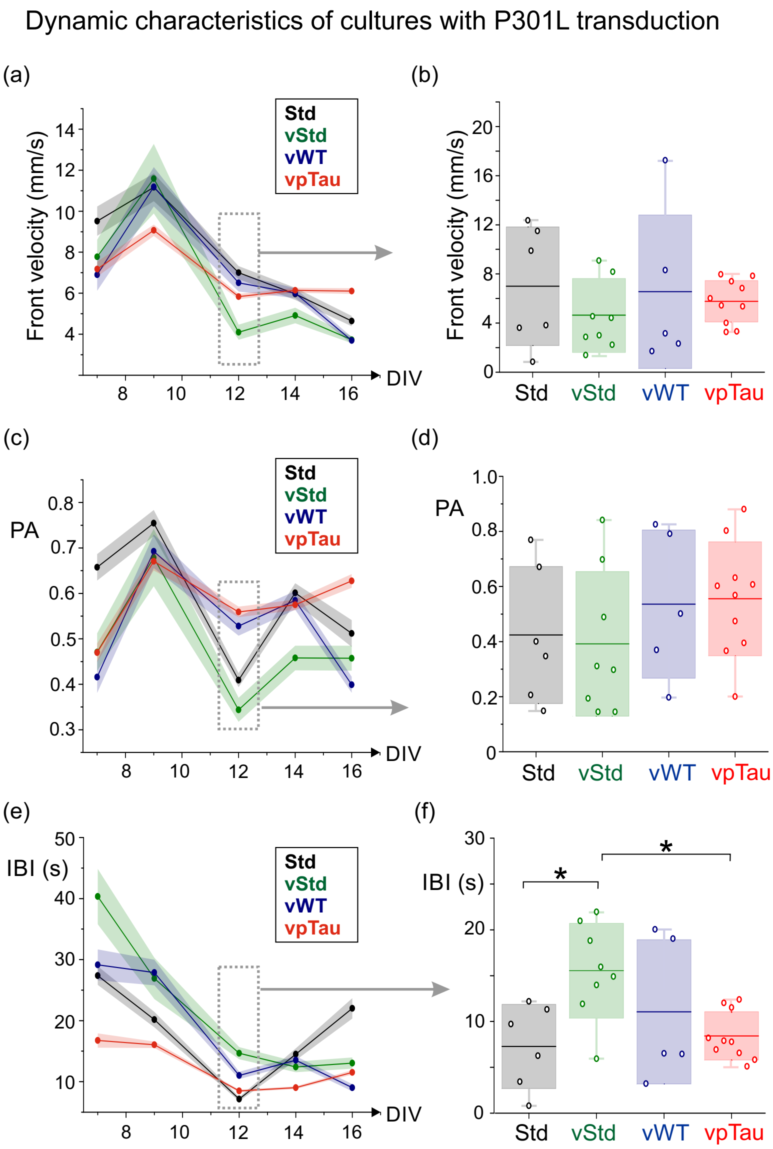

Figure S7. Dynamic descriptors along development in neuronal cultures expressing P301L human tau and subsequently treated with P301S sarkosyl-insoluble fraction. [Related to Figure 11 of the main manuscript]. **(a), (c) and (e)** Evolution along DIV of the average front propagation velocity, population activity and inter-burst interval, respectively, for the four conditions explored (Std, vStd, vWT and vpTau) in 'protocol II'. Each dot is the average value among experimental replicates, and shadings are the standard error. **(b), (d) and (f)** Corresponding box plots for the statistics across experimental replicates and conditions at DIV 12. Colored circles represent independent experimental replicates, horizontal bars indicate the mean, colored boxes the mean ± SD, and whiskers the minimum and maximum values. Sample size is $n = 6$ for Std, $n = 8$ for vStd, $n = 5$ for vWT, and $n = 10$ for vpTau. $^{*}p < 0.05$ (ANOVA followed by Tukey's post-hoc test).

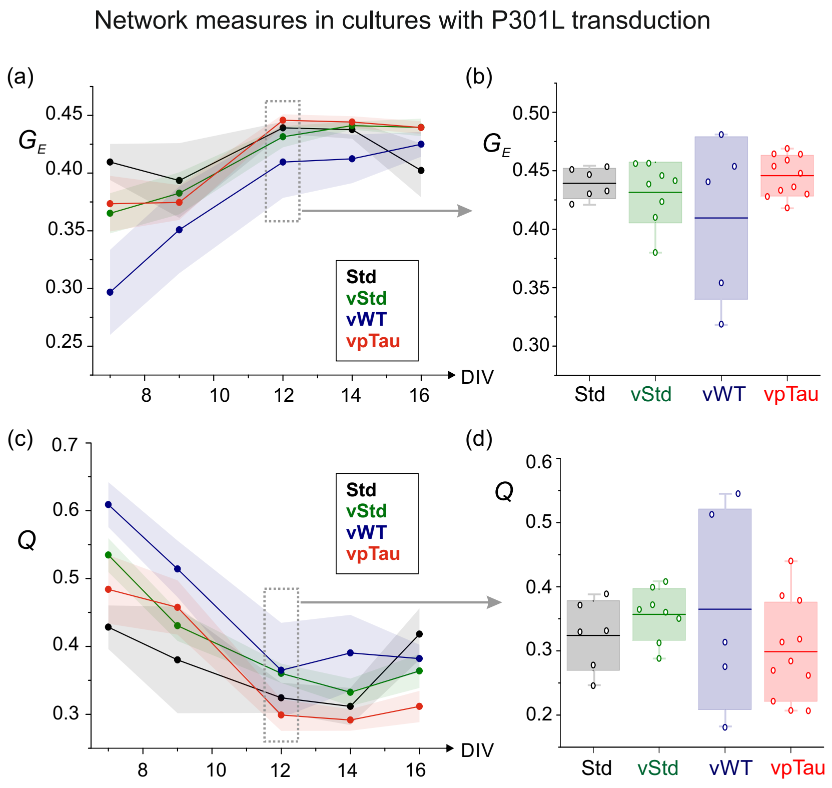

Figure S8. Network metrics along development in neuronal cultures expressing P301L human tau and subsequently treated with P301S sarkosyl-insoluble fraction. [Related to Figure 12 of the main manuscript]. **(a) and (c)** Evolution of average global efficiency G_E and community statistic Q, respectively, along DIV for the four conditions in 'protocol II' (Std, vStd, WT and pTau). Each dot is the average value among experimental replicates, and shadings are standard error. **(b) and (d)** Corresponding box plots for the statistics across experimental replicates and conditions at DIV 12. Colored circles represent independent experimental replicates, horizontal bars indicate the mean, colored boxes the mean ± SD, and whiskers the minimum and maximum values. Sample size is $n = 6$ for Std, $n = 8$ for vStd, $n = 5$ for vWT, and $n = 11$ for vpTau. No statistically significant differences were detected between conditions (ANOVA followed by Tukey's post-hoc test).
